# Sequence Analysis of Novel *Staphylococcus aureus* Lineages from Wild and Captive Macaques

**DOI:** 10.3390/ijms231911225

**Published:** 2022-09-23

**Authors:** Stefan Monecke, Marilyn C. Roberts, Sascha D. Braun, Celia Diezel, Elke Müller, Martin Reinicke, Jörg Linde, Prabhu Raj Joshi, Saroj Paudel, Mahesh Acharya, Mukesh K. Chalise, Andrea T. Feßler, Helmut Hotzel, Laxman Khanal, Narayan P. Koju, Stefan Schwarz, Randall C. Kyes, Ralf Ehricht

**Affiliations:** 1 Leibniz Institute of Photonic Technology (IPHT), 07745 Jena, Germany; 2 InfectoGnostics Research Campus, 07745 Jena, Germany; 3 Institute for Medical Microbiology and Virology, Dresden University Hospital, 01307 Dresden, Germany; 4 Department of Environmental and Occupational Health, School of Public Health, University of Washington, Seattle, WA 98195, USA; 5 Friedrich-Loeffler-Institut (Federal Research Institute for Animal Health), Institute of Bacterial Infections and Zoonoses, 07743 Jena, Germany; 6 Division of Biology, Kansas State University, Manhattan, KS 66506, USA; 7 Nepalese Farming Institute, Maitidevi, Kathmandu 44600, Nepal; 8 Nepal Biodiversity Research Society, Central Department of Zoology, Tribhuvan University, Kirtipur, Kathmandu 44618, Nepal; 9 Institute of Microbiology and Epizootics, Freie Universität Berlin, 14163 Berlin, Germany; 10 Veterinary Centre for Resistance Research (TZR), Freie Universität Berlin, 14163 Berlin, Germany; 11 Central Department of Zoology, Institute of Science and Technology, Tribhuvan University, Kathmandu 44618, Nepal; 12 Center for Postgraduate Studies, Nepal Engineering College, Pokhara University, Lalitpur 33700, Nepal; 13 Department of Psychology, University of Washington, Seattle, WA 98195, USA; 14 Washington National Primate Research Center, Center for Global Field Study, Departments of Psychology, Global Health, Anthropology, University of Washington, Seattle, WA 98195, USA; 15 Institute of Physical Chemistry, Friedrich-Schiller University, 07743 Jena, Germany

**Keywords:** *Staphylococcus aureus*, *Macaca* spp., macaques, next-generation sequencing

## Abstract

*Staphylococcus aureus* is a widespread and common opportunistic bacterium that can colonise or infect humans as well as a wide range of animals. There are a few studies of both methicillin-susceptible *S. aureus* (MSSA) and methicillin-resistant *S. aureus* (MRSA) isolated from monkeys, apes, and lemurs, indicating a presence of a number of poorly or unknown lineages of the pathogen. In order to obtain insight into staphylococcal diversity, we sequenced strains from wild and captive individuals of three macaque species (*Macaca mulatta*, *M. assamensis*, and *M. sylvanus*) using Nanopore and Illumina technologies. These strains were previously identified by microarray as poorly or unknown strains. Isolates of novel lineages ST4168, ST7687, ST7688, ST7689, ST7690, ST7691, ST7692, ST7693, ST7694, ST7695, ST7745, ST7746, ST7747, ST7748, ST7749, ST7750, ST7751, ST7752, ST7753, and ST7754 were sequenced and characterised for the first time. In addition, isolates belonging to ST2990, a lineage also observed in humans, and ST3268, a MRSA strain already known from macaques, were also included into the study. Mobile genetic elements, genomic islands, and carriage of prophages were analysed. There was no evidence for novel host-specific virulence factors. However, a conspicuously high rate of carriage of a pathogenicity island harbouring *edinB* and *etD2*/*etE* as well as a higher number of repeat units within the gene *sasG* (encoding an adhesion factor) than in human isolates were observed. None of the strains harboured the genes encoding Panton–Valentine leukocidin. In conclusion, wildlife including macaques may harbour an unappreciated diversity of *S. aureus* lineages that may be of clinical relevance for humans, livestock, or for wildlife conservation, given the declining state of many wildlife populations.

## 1. Introduction

*Staphylococcus (S.) aureus* is a widespread and common opportunistic bacterium that can colonise or infect humans as well as a wide range of animals [1,2,3,4,5,6,7,8,9,10,11]. Based on well-studied variations affecting sequences of house-keeping genes, a Multilocus Sequence Typing (MLST) scheme has been developed that allows for the unambiguous assignment of isolates to taxonomic categories below species levels, clonal complexes (CCs), and sequence types (STs) [12,13]. This is useful for epidemiological typing but also helps to shed light on the general population structure of the pathogen, and it allows for the description of differences in *S. aureus* populations isolated from different clinical entities, geographic regions, or host species. Although there are a lot of anecdotal reports on *S. aureus* in animals, especially on methicillin-resistant *S. aureus* (MRSA) strains in livestock, there are few studies on wildlife species, and knowledge on *S. aureus* in monkeys and apes is very limited.

There have now been studies of both methicillin-susceptible *S. aureus* (MSSA) and methicillin-resistant *S. aureus* (MRSA) isolated from monkeys, apes, and lemurs from zoos, research centres, wildlife sanctuaries, and the wild [9,14,15,16,17,18,19,20,21,22]. This raises the question of which other, possibly unknown lineages of *S. aureus* might be associated with wild monkeys, including macaques. Such strains might be of clinical relevance for humans, livestock, or, given the declining state of many wildlife populations, wildlife conservation, especially regarding non-human primates. As a result of their evolutionary relationship to humans, it would also be interesting to know if they share *S. aureus* lineages with humans, or if *S. aureus* as a versatile opportunistic pathogen co-evolved with its other host species.

Zoo animals are likely to carry MRSA strains that circulate among humans in the geographic region where the zoo is located. For instance, chimpanzees from an American zoo have been found to harbour the USA300 MRSA strain [16]. Wild animals that have contact with humans might also be colonised by known “human” clones. Various studies in Nepal characterised MRSA isolates from saliva samples collected from wild Rhesus macaques (*Macaca mulatta*) [19,20]. The animals were living in and around temple areas of the Kathmandu valley in Nepal, where human–macaque interaction is common. The most common strain belonged to CC22, a widespread lineage that comprises several related strains with different SCC*mec* IV subtypes and toxin genes profiles [23]. The particular variant found in Nepalese macaques was also observed in humans from the Arabian Gulf region [23] and in Nepalese livestock [24]. These observations led us to hypothesise that humans were a likely source of the CC22-MRSA in the wild Nepalese macaques. The suggestion was that this strain was imported into Nepal possibly by expatriate workers returning from the Gulf states. Livestock might have served as an intermediate host. Some of the other MRSA strains identified could also have epidemiological links to the Middle East. Other studies, in research primate centres, identified CC188-MRSA-IV and ST3268-MRSA-V among macaques of several species imported into the United States [18,21]. CC188-MRSA is found among humans in South-East Asia, but ST3268 appeared to be a “new” clone. The presence of the latter lineage was also observed among captive macaques from Singapore (with ST2817 being a single locus variant of ST3268) [17] and China [22]. Interestingly, it carried an SCC*mec* element apparently very similar to the one in the common “European” livestock-associated MRSA (LA-MRSA) clone (CC398-MRSA-VT) [18]. This suggests that an unknown “monkey-specific” lineage acquired SCC*mec* by co-infection of its macaque hosts with this strain and with CC398 LA-MRSA.

Few studies have yet targeted MSSA from primates. In general, some of the MSSA strains belong to known CCs and might come from humans or from animals/livestock in a specific geographic location. For instance, Nagel et al. [15] found *S. aureus* in lowland gorillas and chimpanzees from a primate facility that presented with *spa* type t148. This type is associated with the MLST CC72, which is a common lineage also in humans. Another study identified strains from wild primates in sub-Saharan African wildlife sanctuaries [14]. Some of these strains clustered with widespread *S. aureus* CCs are found in humans (CC1, CC5, CC8, CC9, CC15, CC30, CC152, and CC188). The study on Nepalese Rhesus and Assam macaques (*Macaca assamensis*) [20] also identified CC15, CC96, and CC2990 MSSA, i.e., strains from lineages that have been observed in humans [25,26,27,28,29,30,31,32].

Some monkey-associated strains belong to the recently described species *S. schweitzeri* and *S. argenteus*. The above-mentioned study [14] identified a large group of deviant isolates (ST1872, ST2022, ST2058, ST2059, and others) belonging to a clade that has been elevated to full species status, *S. schweitzeri*. The second new staphylococcal species, *S. argenteus*, has also been observed in primates, in this case in a wild gorilla from Central Africa [33].

Finally, there are *S. aureus* strains from primates that have not been found in humans or livestock and that might represent native primate lineages, belonging to unique STs not known from humans. In the study by Schaumburg et al. [14], these are ST1928 and ST2023 (ST1728 from this study might be assigned to CC5). Van den Berg et al. [9] identified MSSA strains isolated at the Biomedical Primate Research Centre in The Netherlands from rhesus macaques originating from India, Burma, and China. MLST resulted in 13 novel STs (ST1760, ST1761, ST1768, ST2095, ST2096, ST2097, ST2098, ST2105, ST2106, ST2107, ST2108, ST2119, and ST2120), out of which only two appear to be related to previously known human (ST2108 to CC12) or animal clonal complexes (ST1768 to CC133).

A study on Nepalese Rhesus and Assam macaques [20] identified 30 MSSA isolates that belonged to 18 novel clonal complexes. In the current study, these 18 new clonal complexes, along with ST2990 and two novel MSSA strains isolated from another *Macaca* species, Barbary macaques (*Macaca sylvanus*) from a German zoo, were further characterised using whole-genome sequencing. In addition, a genome sequence of a ST3268-MRSA-VT isolate was analysed as it was, contrarily to previously published sequences of that strain, not fragmented across several contigs.

The aim of this study was to provide full genome sequences of the strains in question and to analyse their phylogenetic relationship to other *S. aureus* lineages, with special regard to emerging strains that might have had a zoonotic background. Furthermore, mobile genetic elements, including genomic islands, pathogenicity islands, prophages, and SCC or SCC*mec* elements were studied with an emphasis on virulence factors and antimicrobial resistance genes.

## 2. Results

Altogether, 22 complete genome sequences were obtained by nanopore and Illumina sequencing (see below) and were analysed (Supplemental Files S1 and S2). A total of two of the study strains belonged to lineages for which genome data were already available (ST2990 and ST3268), 18 have previously been described based on microarray profiles [20], and two of them represented hitherto completely unknown lineages. To give a comprehensive overview, their MLST and phylogeny based on 154 core genomic markers are discussed. Furthermore, the carriage of major genomic islands (GIs) is described, as well as observations regarding the GI-borne adhesion factor *sasG*. Toxin genes, a pathogenicity island with two rare virulence genes (*edinB* and *etD2/etE*), the carriage of bacteriophages, SCC elements, plasmids, and antimicrobial resistance genes are also discussed.

### 2.1. MLST and Phylogeny Based on 154 Core Genomic Markers

**Figure 1:**
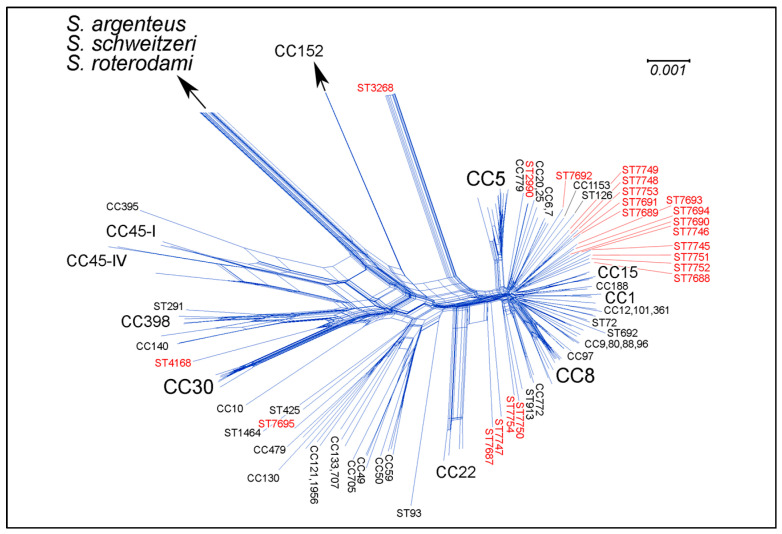
SplitsTree graph [36] based on the sequences of 154 core genomic markers. Study strains are labelled in red. Details on selected sequences and target genes are provided in Supplemental File S3. *S. argenteus*, *S. schweitzeri*, and *S. roterodami* as well as *S. aureus* CC152 are so “distant” that they cannot be included in a figure at this scale (for their relative positions in a comparable graphic representation, see [37], Figure 1).

MLST alleles and STs are summarised in Table 1 along with metadata on geographic origin and host species. An MLST-like approach based on 154 core genomic markers (Figure 1; Supplemental File S3) provides an overview on the position of the study strains relative to other clonal complexes of *S. aureus*. All isolates clearly belonged to *S. aureus* in the narrower sense, i.e., they did not belong to the recently recognised, closely related species *S. argenteus*, *S. schweitzeri* or *S. roterodami*.

Based on core genome markers, *S. aureus* sensu stricto can be divided into three major groups [34], plus some separate branches, as also substantiated by the analysis of array profiles [35]. One major group comprised CC1, CC5, CC8 (Figure 1 and [34,35]), and most of the other *S. aureus* lineages, including CC188, which was previously found in humans and macaques. A second major group (Figure 1 and [34,35]) included human-associated lineages CC59, CC121, and various minor, mostly animal-associated lineages, such as CC49, CC50, CC130, CC133, CC425, CC479, CC599, CC705, CC1464 (“*S. aureus* subsp. *anaerobius*”), and CC1956. A third group consisted of CC10, CC30, CC45, CC140, and CC398 (Figure 1, [34,35]). Separate branches included CC22, CC93, and CC152.

A total of 17 out of 22 macaque CCs (17 out of 19 Nepalese ones) belonged to the first group. Within this group, ST7688, ST7689, ST7690, ST7691, ST7693, ST7694, ST7745, ST7746, ST7748, ST7749, ST7751, ST7752, and ST7753 clustered together. The most closely related non-primate lineage was ST126, a lineage known from cows from Southern Europe [38]. ST7750 and ST7754 clustered with CC772 and CC913, which are clinically important, emerging human lineages from the Indian subcontinent and the Middle East, respectively. ST7692 appeared to be closely related to CC1153, a clonal complex to which MSSA from South-East Asia as well as MRSA from the Middle East belong [39]. The macaque ST2990 isolate was nearly identical with a previously sequenced human isolate of ST2990 from Indonesia (GenBank VCMW; [31]).

The Barbary macaque lineage ST7695 clustered with the second group. The Barbary macaque lineage ST4168 belonged to the third group. The macaque-associated MRSA strain, ST3268 [18,21], constituted an additional, clearly separate branch. Macaque CCs ST7687 and ST7747 formed another one or two separate branches, being closer to CC22 than to others.

### 2.2. Description of the Clonal Complexes and Their Genomic Islands

Clonal complexes of *S. aureus* can be discerned not only based on MLST alleles and alleles of other, conserved, or housekeeping genes (as used for the construction of the phylogenetic tree, see Figure 1 and Supplemental File S3a,b) but also on the presence of a number of genomic islands (GIs, with GI being defined as “large genomic regions that are found in bacterial genomes and that have probably been horizontally acquired” [40]). The regulatory *agr* gene cluster and the capsule operon could also be regarded as GIs. The affiliations to *agr* groups and capsule types are provided together with the MLST profiles in Table 1. SCC/SCC*mec* elements and pathogenicity islands that, strictly speaking, also fulfill the definition of a GI are discussed separately (see below).

We analysed a total of 15 important, well-characterised, and/or CC-associated GIs (but a variety of smaller GIs, or those with variable positions within the genome, were not analysed, so the following listing is not an exhaustive summary of GIs in *S. aureus*). The analysed GIs inhabit positions within the genome that are conserved among all CCs that carry the respective GI. Their carriage is (usually) uniform among all strains belonging to a given CC, but similar/identical GIs might appear in phylogenetically unrelated lineages. An abridged overview on the carriage of these 15 “major GIs” by the study isolates is shown in Table 2, and the full list of genes in the respective GIs is provided in Supplemental File S4. These “major GIs” include the following ones.

1. GI between SCC integration site (*orfX*) and *dusC* around position 38,000 in the genome. This is the GI that harbours the enterotoxin homologue ORF CM14 in CC93, CC121, and CC772, or the enterotoxin gene *seh* in CC1, but these particular genes were absent from all study strains. There are about 40–45 genes or putative genes associated with this island, out of which zero to 15 can be found in any *S. aureus* sequence, present in CC-specific combinations and usually in a conserved sequential arrangement. In addition, there could be some transposase genes. All study strains but two carried a GI in this position, and twelve variants could be distinguished
2. GI immediately downstream of *dusC.* There are about 25 genes or putative genes associated with this position, and different CCs of *S. aureus* carry 0 to 16 (in CC45) of them, again in CC-specific patterns and conserved order. All study strains harboured a GI in this position, and there were ten distinct variants consisting of one to twelve genes.
3. GI with *lpl* genes (tandem lipoprotein genes), position 40–80,000. Strains harboured varying numbers of *lpl* gene copies, in some cases accompanied by *lipC3* (putative lipase class 3) and *hysA* (hyaluronate lyase) genes.
4. GI adjacent to the first *opp*-operon, approx. position 170,000. All strains carried a GI in this position, and there were five distinct variants. The most common one (consisting of 3 genes) can also be observed in the CC8 sequence of COL (CP000046.1). In three strains, only the first of these genes was present, being truncated as in the CC30 strain MRSA-252 (BX571856.1). In one strain, this gene alone was detected followed by 2 transposase genes. In two strains, a 10-genes ABC transporter operon was found as in CC705 RF122 (AJ938182.1), although in one of them, this was followed by yet another gene (Table 2/Supplemental File S4).
5. GI around position 280,000. In all strains, this position was occupied by a GI. Discounting the variability of the copy number of the DUF600 gene for a “putative protein”, sixteen different variants were identified. Four variants, in eight strains, included the *esxC*/*esxB*/*esaE*/*esxD*/*essD* gene cluster apparently associated with virulence [41]. Note that the adjacent and related genes around *esxA* were, contrary to the *esxB* cluster, always present and thus considered as a core genome. In one strain, ST3268-MRSA-VT, this island served as an integration site for a transposon carrying the beta-lactamase operon (*blaZ*/*I*/*R*).
6. GI with *ssl* (staphylococcal superantigen like protein) and *lpl* genes around position 400,000, consisting of *ssl01* to *ssl10*, a restriction–modification system *hsdM*/*S-ssl*, *ssl11,* and *slap*, followed by a variable number of *lpl* genes and *lipC3* (putative lipase class 3). It was always present, although several strains lacked the *ssl06* gene (by array as well as by sequencing, ruling out annotation artefacts) or *lpl*/*lipC3* genes.
7. GI adjacent to the second *opp*-operon, approx. position 900,000. This position is occupied by genes encoding oligopeptide ABC transporter systems. One variant, *oppB*/*C*/*D*/*F*/*A*-GI, is related to sequences from CC93, CC398, and CC705. It was present in two of the lineages (ST4168 and ST7748). The other variant is *appA*/*D*/*F*/*B*/*C*, being related to the corresponding GI in CC1, CC5, CC8, or CC22. This one was present in the other twenty strains.
8. GI carrying ABC transporter/bacteriocin genes, position 1,500,000. Six lineages (Table 2) harboured a GI with *sagC*/*D* genes encoding a bacteriocin biosynthesis operon, an ABC transporter system (Q2YYD5, D9RHF8) and *stsA*, encoding a stapholysin-like polypeptide (AJ938182.1 (1,504,962 to 1,505,114)). Four had an apparently truncated variant thereof that lacked *sagC*/*D*. Two carried only Q931R4 (major facilitator superfamily transporter as in CC5, CC15, CC30, CC97, and CC188) instead, and in ten strains, this position was not occupied by any GIs.
9. GI around position 1,800,000. This is a large GI consisting of a variable cast of genes, including a restriction–modification system *hsdM*/*S-spl*, several serine proteases (*spl* genes), the enterotoxin gene cluster *egc* (*seg*, *sei*, *selm*, *seln*, *selo*, *selu*), the leukocidin genes *lukD*/*E*, a lantibiotic epidermin biosynthesis cluster (*epi* genes), a putative bacteriocin (*bsaX*), and several genes encoding “putative proteins”. All strains harboured a GI in this position. All of them included *lukD*/*E*, and the *egc* enterotoxin gene cluster was present in six (see below).
10. GI carrying *sspP*/*sspS*, staphopain A/staphostatin A genes, position 1,950,000 to 2,000,000. This island appears to be present in all *S. aureus* strains and in at least some *S. schweitzeri* but is absent from *S. argenteus* and *S. roterodami*. It was detected in all study strains.
11. GI with *lpl* genes, position 2,500,000. This island usually consists of a non-coding RNA (as in N315, BA000018.3 (2,544,997 to 2,545,070); “SAU-19”, see [42]), a variable number of *lpl* genes, *lipC3*, some genes encoding “putative proteins”, and a type II restriction–modification system (TII-RM; endonuclease plus methyltransferase). For the latter, five alleles that strictly correlate with CC affiliation are distinguished (sau3AI; M32470.1 as in CC9/15/121/188; sau96I; X53096.1 as in CC25; sauRF122; AJ938182.1 as in CC705; sauS0385; AM990992.1 as in CC398 and sauUSI; CP000046.1 as in most other CCs, including CC1/5/8/30). All these five variants were found among the study strains (Table 2/Supplemental File S4).
12. GI carrying *sasG*, around position 2,530,000. Eighteen out of 22 strains carried a GI in this position, but only eleven were positive for the *sasG* gene. This gene is discussed separately (see below).
13. Staphyloxanthin gene cluster, around position 2,650,000, consisting of *crtN*, *crtM*, *crtQ*, *crtP*, and *crtO*. It is known to be absent from *S. argenteus* and *S. aureus* CC152, whereas deviant alleles can be observed in *S. roterodami*, *S. schweitzeri*, and *S. aureus* CC93. This cluster was present in all isolates, and none of the known deviant alleles were identified.
14. Cobalt transporter GI, around position 2,770,000. This GI invariably consists of *cbiQ*/*O* (putative cobalt ABC transporter, transmembrane permease, and ATP-binding protein) and genes encoding a transmembrane protein and an adenosyltransferase. It was present in twenty strains.
15. GI carrying the collagen adhesin gene *cna*, around position 2,780,000. This gene was detected by array hybridisation as well as by sequence analyses in ten out of 22 strains.

**Table 2:** Major genomic islands in study strains (please note that this is an abridged table; the full set is provided in Supplemental File S4).

Sequence Type, (Isolate ID)	GI No. 1 Upstream *dusC*, Approx. Pos. 38,000	GI No. 2 Downstream *dusC*, Approx. Pos. 38,000	GI No. 3, Approx. Pos. 40–80,000	GI No. 4, Approx. Pos. 170,000	GI No. 5, Approx. Pos. 280,000	GI No. 6 (*ssl*/*lpl*), Approx. Pos. 400,000	GI No. 7, Approx. Pos. 900,000	GI No. 8, Pos. 1,500,000	GI No. 9 (e*gc* and *lukD*/*E*) Approx. Pos. 1,800,000	GI No. 10 (*sspP*/*sspS*), Approx. Pos. 2,000,000	GI No. 11, Approx. Pos. 2,500,000	GI No. 12, Approx. Pos. 2,530,000	GI No. 13 (staphyloxanthin), Approx. Pos. 2,650,000	GI No. 14, Approx. Pos. 2,770,000	GI No. 15 (*cna*), Approx. Pos. 2,780,000
ST2990 (27-G-H)	As in CC5, CC8, CC9	Q6GKK6, Q7A890	*lpl*, *hysA*, 5 copies of *lpl*	As in COL (CC8)	Present, includes a 2^nd^ copy of *essC*	Present, but lacks *lpl*/*lipC3* genes	*app*-operon	Absent	Present, includes *hsdS*/*M-spl*, *splF*/*E*/*D1*/*D2-1*/*D2-2*/*C*/*A*, *epiG*, *lukD*/*E*	*sspP*/*sspS*	ncRNA, 3 copies of *lpl*, *sau*USI	Present, includes *sasG* (CC1-like allele with 8 ½ repeats)	Present	Present	Present
ST3268 (Ma2/A14043)	Unique Pattern	Q6GKK6, ycjY, G7ZTC1, Q2YUT3	3 copies of *lpl*	Truncated as in MRSA252 (CC30)	Transposon with the penicillinase operon	Present, but *lipC3* replaced by transposase gene	*app*-operon	Present, includes *sagD*/*C* and *stsA*	Present, includes *hsdS*/*M-spl*, *epiG*, *lukD*/*E*, *seg*/*n*/*u*/*i*/*m*/*o*	*sspP*/*sspS*	ncRNA, *sau*96I,	Present, includes *sasG* (CC5/8-like allele with 7 ½ repeats)	Present	Present	Absent
ST4168 (16CS0209)	Unique Pattern	As in ST2990	*lpl*	Truncated as in MRSA252 (CC30)	Present	Present, but lacks *ssl06*	*opp*-GI-operon	Q931R4	Present, includes *hsdS*/*M-spl*, *splF*/*E*/*D2*/*C*/*B*/*A*, *epiG*/*F*/*D*/*C*/*B*/*A*, *lukD*/*E*, *seg*/*n*/*u*/*i*/*m*/*o*	*sspP*/*sspS*	ncRNA, followed by approx. 3000 nt insert	Present, includes *sasG* (CC5/8-like allele with 9 ½ repeats)	Present	Present	Present
ST7687 (01-RR-86)	As in CC5, CC8, CC9	As in ST2990	3 copies of *lpl*	As in COL (CC8)	Present	Present	*app*-operon	Present, includes *sagD*/*C* and *stsA*	Present, includes *hsdS*/*M-spl*, *splF*/*C*/*B*/*A*, *epiG*, *lukD*/*E*	*sspP*/*sspS*	ncRNA, 2 putative proteins, *lipC3*, 3 copies of *lpl*, *sau*S0385	Present, includes *sasG* (CC5/8-like allele with 12 ½ repeats)	Present	Present	Absent
ST7688 (05-RR-90)	As in CC5, CC8, CC9	10-gene pattern	3 copies of *lpl*	As in COL (CC8)	Present	Present, but lacks *ssl06*	*app*-operon	Present, includes *stsA*	Present, includes *hsdS*/*M-spl*, *splF*/*E*/*D1*/*C*/*B*/*A*, *epiG*, *lukD*/*E*	*sspP*/*sspS*	ncRNA, 4 copies of *lpl*, 2 putative proteins, *lipC3*, *sau*3AI	Present, includes *sasG* (CC1-like allele with 9 ½ repeats)	Present	Present	Absent
ST7689 (08-G-E)	1 gene only (Q6GD44)	As in ST2990	2 copies of *lpl*, *lipC3*	As in COL (CC8)	Present, includes *esxC*, *esxB*, *esaE*, *esxD*, *essD*	Present, but lacks *ssl06*	*app*-operon	Absent	Present, includes *hsdS*/*M-spl*, *splF*/*D1*/*C*/*B*/*A*, *lukD*/*E*, *seg*/*n*/*u*/*i*/*m*/*o*	*sspP*/*sspS*	ncRNA, 5 copies of *lpl*, 2 putative proteins, *lipC3*, *sau*USI	Present, includes *sasG* (CC5/8-like allele with 6 ½ repeats)	Present	Present	Absent
ST7690 (09-G-F)	As in CC5, CC8, CC9	10-gene pattern as in ST7688 plus 2 *tnp* copies	3 copies of *lpl*, *hysA*, 3 copies of *lpl*	Q5HJH7 + 2 *tnp*	Present	Present, but lacks *ssl06*	*app*-operon	Absent	Present, includes *hsdS*/*M-spl*, *splF*/*E*/*C*/*B*/*A*, *epiG*, *lukD*/*E*	*sspP*/*sspS*	ncRNA, 5 copies of *lpl tnp*, 2 putative proteins, *lipC3*, *sau*S0385	Present, includes *sasG* (CC5/8-like allele with 8 ½ repeats)	Present	Present	Present
ST7691 (13-G-52)	As in ST7689	As in ST2990	2 copies of *lpl*, *lipC3*	As in COL (CC8)	Present, includes *esxC*, *esxB*, *esaE*, *esxD*, *essD*	Present	*app*-operon	Absent	Present, includes *hsdS*/*M-spl*, *splF*/*D1*/*C*/*B*/*A*, *lukD*/*E*, *seg*/*n*/*u*/*i*/*m*/*o*	*sspP*/*sspS*	ncRNA, 4 copies of *lpl*, 2 putative proteins, *lipC3*, *sau*USI	Present, includes *sasG* (CC5/8-like allele with 10 ½ repeats)	Present	Present	Present
ST7692 (17-H-61)	As in ST7689	10-gene pattern as in ST7688	4 copies of *lpl*	As in COL (CC8)	Present	Present, but lacks *ssl06*	*app*-operon	Present, includes *sagD*/*C* and *stsA*	Present, includes *hsdS*/*M-spl*, *splF*/*E*/*D1*/*C*/*B*/*A*, *epiG*, *lukD*/*E*	*sspP*/*sspS*	ncRNA, 6 copies of *lpl*, 2 putative proteins, *lipC3*, *sau*3AI	Present, includes *sasG* (CC5/8-like allele with 12 ½ repeats)	Present	Present	Absent
ST7693 (29-P-01)	Absent	5-gene pattern	*lpl*	As in COL (CC8)	Present, includes *esxC*, *esxB*, *esaE*, *esxD*, *essD*	Present, but lacks *ssl06*	*app*-operon	Absent	Present, includes *hsdS*/*M-spl*, *splF*/*E*/*D1*/*C*/*B*/*A*, *lukD*/*E*	*sspP*/*sspS sspP*/*sspS*	ncRNA, 2 putative proteins, *lipC3*, *lpl*, *sau*RF122	Present, without *sasG*	Present	Present	Absent
ST7694 (40-B-50)	Absent	5-gene pattern as in ST7693	5 copies of *lpl*	Truncated as in MRSA252 (CC30)	Present, includes *esxC*, *esxB*, *esaE*, *esxD*, *essD*	Present, but lacks *ssl06* and *lpl*/*lipC3* genes	*app*-operon	Present, includes *stsA*	Present, includes *hsdS*/*M-spl*, *splF*/*D1*/*C*/*B*/*A*, *epiG*, *lukD*/*E*	*sspP*/*sspS*	ncRNA, 4 copies of *lpl*, *sau*USI,	Present, includes *sasG* (CC1-like allele with 14 ½ repeats)	Present	Present	Absent
ST7695 (16CS0212)	1 gene only (Q6GKL6)	10-gene pattern as in ST7688	*lpl*	As in COL (CC8)	Present, includes *esxC*, *esxB*, *esaE*, *esxD*, *essD*	Present, but lacks *ssl06*	*app*-operon	Absent	Present, includes *hsdS*/*M-spl*, *splF*/*D2*/*E*/*C*/*B*/*A*, *epiG*/*E*/*F*/*P*/*D*/*C*/*B*/*A*, *bsaX*, *lukD*/*E*	*sspP*/*sspS*	ncRNA, 3 copies of *lpl*, 2 putative proteins, *lipC3*, *sau*3AI	Absent	Present	Absent	Absent
ST7745 (03-RR-88)	Unique Pattern	As in ST2990	5 copies of *lpl*	As in COL (CC8)	Present	Present	*app*-operon	Present, includes *sagD*/*C* and *stsA*	Present, includes *hsdS*/*M-spl*, *splF*/*E*/*C*/*B*/*A*, *epiG*, *lukD*/*E*	*sspP*/*sspS*	ncRNA, 2 putative proteins, *lipC3*, *lpl*, *sau*RF122	Absent	Present	Present	Present
ST7746 (07-G-D)	As in CC5, CC8, CC9	As in ST7695 plus Q7A890	6 copies of *lpl*	As in RF122 (CC705) plus A6QDI2	Present	Present, but lacks *lpl*/*lipC3* genes	*app*-operon	Absent	Present, includes *hsdS*/*M-spl*, *splF*/*E*/*D1*/*C*/*B*/*A*, *epiG*, *lukD*/*E*	*sspP*/*sspS*	ncRNA, 6 copies of *lpl*, 3 putative proteins, 2 copies of *lipC3*, *tnp*, *sau*S0385	Absent	Present	Present	Present
ST7747 (12-G-51)	As in CC398	As in ST2990	*lpl*, *hysA*, 2 copies of *lpl*, *istB2*_IS232, *tnp*, *lpl*	As in COL (CC8)	Present	Present, but lacks *ssl06*	*app*-operon	Present, includes *sagD*/*C* and *stsA*	Present, includes *hsdS*/*M-spl*, *splF*/*E*/*D1*/*C*/*B*/*A*, *epiG*/*E*/*F*/*P*/*D*/*C*/*B*/*A*, *bsaX*, *epiA*, *lukD*/*E*	*sspP*/*sspS*	ncRNA, 2 copies of *lpl*, 2 putative proteins, *lipC3*, *sau*S0385	Present, without *sasG*	Present	Absent	Absent
ST7748 (15-G-54)	As in ST7689	As in ST2990	2 copies of *lpl*, *lipC3*	As in COL (CC8)	Present, includes *esxC*, *esxB*, *esaE*, *esxD*, *essD*	Present, but lacks *ssl06*	*opp*-GI-operon	Absent	Present, includes *hsdS*/*M-spl*, *splF*/*E*/*D1*/*C*/*B*/*A*, *lukD*/*E*, *seg*/*n*/*u*/*i*/*m*/*o*	*sspP*/*sspS*	ncRNA, 3 copies of *lpl*, 2 putative proteins, *lipC3*, *sau*3AI	Present, without *sasG*	Present	Present	Present
ST7749 (18-H-62)	Unique Pattern	As in ST2990	3 copies of *lpl*, *hysA*, 3 copies of *lpl*	As in COL (CC8)	Present	Present, but lacks *ssl06*	*app*-operon	Present, includes *stsA*	Present, includes *hsdS*/*M-spl*, *splF*, *splC*/*B*/*A*, *epiG*/*E*/*F*/*P*/*D*/*C*/*B*/*A*, *lukD*/*E*	*sspP*/*sspS*	ncRNA, 4 copies of *lpl* 2 putative proteins, *lipC3*, *sau*3AI	Present, without *sasG*	Present	Present	Absent
ST7750 (26-G-G)	Unique Pattern	Q6GKK6	3 copies of *lpl*, *lipC3*	As in RF122 (CC705)	Present	Present, but lacks *ssl06*	*app*-operon	Present, includes *sagD*/*C* and *stsA*	Present, includes *hsdS*/*M-spl*, *splF*/*E*/*C*/*B*/*A*, *epiG*, *lukD*/*E*	*sspP*/*sspS*	ncRNA, 4 copies of *lpl* 2 putative proteins, *lipC3sau*3AI	Present, includes *sasG* (CC1-like allele with 9 ½ repeats)	Present	Present	Present
ST7751 (28-G-I)	Unique Pattern	4 genes as in CC1153	*lpl*, *hysA*, 4 copies of *lpl*	As in COL (CC8)	Present, includes *esxC*, *esxB*, *esaE*, *esxD*, *essD*	Present, but lacks *ssl06* and *lipC3*	*app*-operon	Absent	Present, includes *hsdS*/*M-spl*, *splF*/*E*/*D1*/*C*/*B*/*A*, *epiG*, *lukD*/*E*	*sspP*/*sspS*	ncRNA, 4 copies of *lpl*, 2 putative proteins, *lipC3*, *sau*3AI	Present, without *sasG*	Present	Present	Absent
ST7752 (30-P-10)	As in CC5, CC8, CC9	As in ST7695	5 copies of *lpl*	As in COL (CC8)	Present	Present	*app*-operon	Absent	Present, includes *hsdS*/*M-spl*, *splF*/*E*/*D1*/*C*/*B*/*A*, *epiG*, *lukD*, *lukE*	*sspP*/*sspS*	ncRNA, *lpl*, *sau*3AI	Present, without *sasG*	Present	Present	Absent
ST7753 (32-T-13)	Unique Pattern	As in ST2990	6 copies of *lpl*	As in COL (CC8)	Present, includes *esxC*, *esxB*, *esaE*, *esxD*, *essD*	Present, but lacks *ssl06* and *lipC3*	*app*-operon	Present, includes *stsA*	Present, includes *hsdS*/*M-spl*, *splF*/*D1*/*C*/*B*/*A*, *lukD*/*E*, *seg*/*n*/*u*/*i*/*m*/*o*	*sspP*/*sspS*	ncRNA, 2 putative proteins, *lipC3*, *lpl*, *sau*RF122	Present, without *sasG*	Present	Present	Present
ST7754 (39-B-49)	Unique Pattern	As in ST2990	*lpl*, *lipC3*	As in COL (CC8)	Present	Present, but lacks *ssl06*	*app*-operon	Q931R4	Present, includes *hsdS*/*M-spl*, *splF*/*E*/*D1*/*C*/*B*/*A*, *epiG*, *lukD*/*E*	*sspP*/*sspS*	2 putative proteins, *lipC3*, 2 copies of *lpl*, *sau*USI,	Absent	Present	Present	Present

### 2.3. The sasG Gene

**Figure 2:**
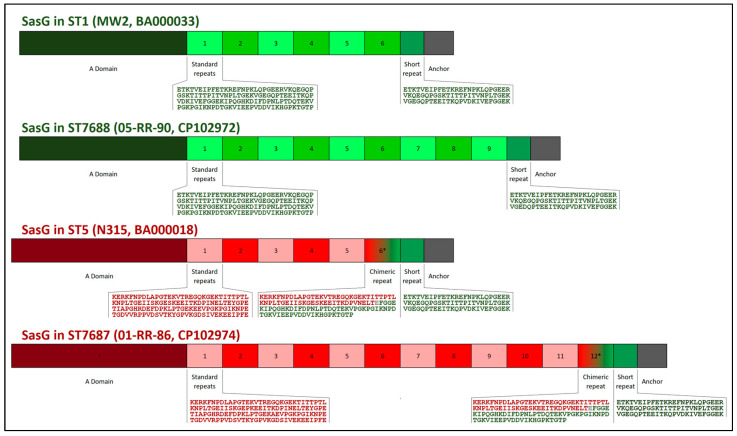
Diagram of *S. aureus* surface protein G comparing the two different alleles and different repeat numbers.

As shown in Table 2, the *sasG* gene, a genomic island-borne gene for *S. aureus* surface protein G, was present in 11 out of 22 lineages. An interesting observation was the length of this gene. It was longer than *sasG* from published sequences of isolates mainly derived from humans [43], with an average length among the *sasG*-positive study strains of about 5442 nt (median, 5268 nt; range, 4116 to 7188 nt). This difference was related to the number of the repeat units this gene comprises, translating into a presence of 9 full repeats (median; ranging from 6 to 14 repeats; Table 2; Figure 2) plus 1 additional, truncated terminal repeat (referred to as “½ repeat” in Table 2, see also below). A recent work analysed 353 *sasG* sequences identified in GenBank from full genomes mostly of human strains [43]. These contained a median of only 3 full repeats (range from 0 to 9 repeats), plus the 1 truncated terminal repeat.

Previously described sequence data indicate that there are two main variants or alleles of *sasG* (Figure 2). One can be found in CC1 and a number of sporadic lineages. The other one is present in CC5 and CC8 [43]. They can be discerned based not on the numbers, which are variable, but on the actual sequence of the repeat units and on the sequence of the “A domain” of the deduced protein [43]. In the CC1-like allele, lengths (128 amino acids, aa) and sequences of all repeats are uniform, except for the last one, adjacent to the anchor, which is a shorter, truncated version of the others (75 aa rather than 128 aa; Figure 2). In the CC5/8 allele, most repeats are of equal length (128 aa) but have a different sequence. However, the pre-terminal repeat is shorter (118 aa), and it appears to be a chimera comprising a CC5/8-like part of 59 or 60 aa and a CC1-like part of 58 or 59 aa (the amino acid in pos. 60, E, could be of either origin). The last repeat, adjacent to the anchor, has the same sequence as the one in the CC1-like allele of *sasG*, and the anchor sequence is also conserved in both alleles. All macaque isolates that harboured *sasG* were assignable to either of these two variants, with four of them (ST2990, ST7688, ST7694, and ST7750) matching the CC1-like allele, and the other seven *sasG*-positives carried the same allele as present in CC5/CC8.

### 2.4. Toxin Genes

All clonal complexes carried the enterotoxin homologue “*entX*” (corresponding SACOL1657), and it was always localised at the same position in the respective genomes, around 1,600,000. However, in ST7693 (29-P-01), a fragment of 225 nt appeared to be duplicated within the gene’s sequence. The staphylococcal enterotoxin-like toxin X, *selX*/*setC* (SACOL0442), was present in all clonal complexes except ST4168.

Six clonal complexes (ST3268, ST4168, ST7689, ST7691, ST7748, and ST7753; see also Table 2 and above, in the paragraph on genomic islands) carried the enterotoxin gene cluster *egc* (consisting of *seg*, *sei*, *selm*, *seln*, *selo*, and *selu*). Enterotoxin genes *sec* and *sel* were found in two isolates. The ST2990 isolate carried them as part of a pathogenicity island localised between *guaA* and Q2YVN4 (corresponding SACOL0461 and SACOL0465), accompanied by an integrase gene related to the one from mobile pathogenicity island SaPIbov1 and by *ear* (encoding a putative “enterotoxin-linked ampicillin resistance protein” frequently associated with these enterotoxin genes). The ST3268-MRSA-VT isolate also carried these two enterotoxin genes, on a similar element at the same location that, however, additionally harboured an aminoglycoside resistance gene (see below). No other enterotoxin genes were identified, neither by microarray nor by sequencing.

All isolates of all CCs carried the gamma leukocidin locus (*lukF*/*S-hlg*, *hlgA*) as well as leukocidin genes *lukD*/*E* (see Table 2) and *lukA*/*B* (=*lukG*/*H* or *lukX*/*Y* [35,44,45,46]). The phage-borne leukocidin *lukS*/*F-PV* (encoding Panton–Valentine leukocidin) as well as animal-associated related genes *lukM*/*lukF-P83*, *lukP*/*Q* [47], and *lukS*/*F*-*BV* [48] were absent from all study strains, although *lukS*/*F-PV* was previously observed in human isolates of CC2990 (author’s unpubl. observation). Exfoliative toxin genes *etA*, *etB*, and *etD* as well as epidermal cell differentiation inhibitor genes *edinA* and *edinC* were not identified. However, *etD2*/*etE* and *edinB* were present in five clonal complexes. This is discussed in the next paragraph.

### 2.5. The Pathogenicity Island Carrying edinB and etD2/etE

Five of the macaque-associated clonal complexes harboured a pathogenicity island carrying *edinB* and *etD2*/*etE* (ST2990, ST7687, ST7690, ST7749, ST7750). In size, gene content, and localisation, it was very similar to a pathogenicity island in CC130 (with the CC130-MSSA strain O11, CP024649.1 being used here for comparison and reference; see Table 3).

**Figure 3:**
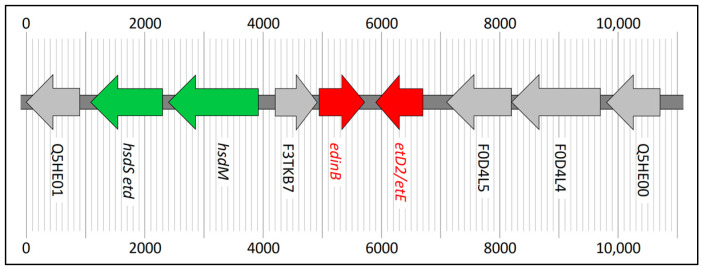
The pathogenicity island with *edinB* and *etD2*/*etE* as present in ST2990, ST7687, ST7749, ST7750, and CC130 (see also Table 3).

In all macaque strains, it is integrated between a hyaluronate lyase gene (corresponding to CP024649.1 (2,245,391 to 2,247,811), SaO11_02005) and an extracellular adherence protein gene homologue (corresponding to CP024649.1 (2,258,831 to 2,259,256), SaO11_02015) at approximately position 2,200,000 of the genome. It was about 11,000 bp long, and it consisted, as it does in CC130 (Strain O11, GenBank CP024649.1), of the genes shown in Table 2 and Figure 3. In one strain (ST7690), genes for a transposase and a transposase helper protein were integrated that were absent from the others as well as from the reference sequence CP024649.1. Another strain (ST2990) had a deletion of about 100 nt affecting the gene encoding Q5HE01. The *hsdS* genes presented, despite uniform length, with two different alleles: one in ST7687 and ST7749 and the other one in ST2990, ST7690, and ST7750. CC130 harboured a third allele of that gene. Apart from these differences, the pathogenicity island sequences of the macaque strains were nearly identical, differing only in few single nucleotide polymorphisms (SNPs). Regarding these SNPs, ST7687 and ST7749 (18-H-62) clustered together and with the CC130 sequence.

### 2.6. Carriage of Prophages

The carriage of prophages and their integration sites are summarised in Table 4. Only one isolate carried an *hlb*-converting prophage, and this was the one assigned to ST2990. Its sequence included *sak* (staphylokinase), *scn* (staphylococcal complement inhibitor), and *chp* (chemotaxis-inhibiting protein CHIPS). In addition, there were another eight phage integration sites, out of which six were inhabited by prophages that could be suspected to Siphoviridae based on sequence similarities to known phages.

One integration site, between *glnA* (=*femC*) and A6U1C8 (SACOL1329 and SACOL1331), around position 1,300,000, harboured phage-specific genes, but identification was considered not safe due to a small number of identified genes, most of which originated from an *S. schweitzeri* genome sequence (CCEL01000004.1). A few genes further downstream, between Q2YXQ4 (SACOL1335) and A6QGL8 (SACOL1349), around position 1,300,000, there was another integration site that in several strains was occupied by genes related to capsid genes annotated elsewhere. None of the phages contained known phage-borne enterotoxin or leukocidin genes (*sea* and its alleles, *see*, *lukF*/*S-PV*, *lukF*/*S-BV*, *lukM*/*lukF-P83*, and *lukP*/*Q*).

### 2.7. Carriage of SCC Elements and Associated Genes

As expected, based on array analysis, the ST3268 isolate carried a SCC*mec* VT element. It was nearly identical to the one in the European LA-MRSA strain CC398-MRSA-VT. A direct comparison to its reference sequence AM990992 is shown in Table 5. The only difference was the presence of the tetracycline resistance *tet*(K) that appeared to be located on a small plasmid integrated via the insertion sequence IS*431* into the SCC*mec* element of the ST3268 isolate.

None of the other strains discussed carried *mecA*, *mecC*, *fusc*, or *ccrA*/*B* recombinase genes, but eight lineages harboured, directly downstream of *orfX*, other genes known to be associated with SCC elements. Five strains, namely ST7689, ST7692, ST7691, ST7748, and ST7753, were found by array as well as by sequencing to carry a gene (CP003979.1 (59,396 to 60,196), SAKOR_00054) encoding B2Y834, an abortive phage resistance protein that is associated with SCC*mec* IV A, SCC*mec* IV G, SCC*mec* IVc, and SCC*mec*-MRSAZH47, as well as with SCC elements without *mecA*/*C* in CC188. In all study strains, it was localised directly (309 or 378 nt) downstream of *orfX.* Another strain, ST7694 (40B50), harboured a gene for a putative protein F4NA83 followed by two different variants of *dhlC* (a DNA helicase gene) and a transposase gene. This is a constellation similar to CC130, including both, methicillin susceptible strains (CP024649.1), as well as CC130-MRSA-XI (FR823292.1). A similar element was found in ST7693 (29P01), but this one included only one copy of a helicase gene. Finally, ST7745 carried another SCC*mec*-associated gene, encoding a putative protein Q5HK75 (AJLX01000030 (23,381 to 25,201)).

### 2.8. Carriage of Plasmids

Three strains, ST7688, ST7691, and ST7694 carried four plasmids or plasmid-associated contigs, p05RR90 (ST7688), p13G52 (ST7691), p40B50-ctg2, and p40B50-ctg3 (ST7694). Two of them harboured cadmium resistance genes *cadD* (cadmium transport protein D; p13G52, identical to Newbould_305, AKYW01000028 (1556 to 2173)) and *cadX* (putative regulator of cadmium efflux; p13G52, identical to Newbould_305, AKYW01000028 (1190 to 1537)); p40B50-ctg2, identical to AY373761 (1049 to 1396)).

### 2.9. Carriage of Other Resistance Genes

The ST3268 isolate carried, in addition to the SCC*mec* element, also a beta-lactamase operon (*blaZ*/*R*/*I*). It was present on a transposon integrated into the strain’s genome, namely into the GI usually associated with the virulence gene *esxB* (see Table 2).

This strain also harboured the aminoglycoside 6-adenyltransferase gene *aadK*, localised together with an integrase gene, *ear* and enterotoxin genes *sec* and *sel* on a pathogenicity island located between *guaA* and Q2YVN4. In ST4168, ST7688, and ST7691, the same position was occupied by other mobile genetic elements that also included *aadK*. Other genes known to be associated with antimicrobial resistance were not found.

The ubiquitous chromosomal genes associated with heavy metal resistance properties *arsB* (arsenical pump membrane protein) and *arsR* (repressor of arsenic resistance operon) as well as *czrB=zntA* (zink and cobalt transporter protein) and its regulator *czrA=zntR* were found in all strains. An additional arsenic resistance gene, chromosomal *arsC*, was present in all lineages except for ST3268, ST4168, ST7687, ST7690, ST7692, ST7695, and ST7747.

## 3. Discussion

One result of the present study is that the biological diversity even of well-known, easily culturable opportunistic pathogens, such as *S. aureus*, in wildlife is still underappreciated. A comparatively small sample of animal strains, from just three species (Rhesus, Barbary and Assam macaques), yielded as many as twenty different “new” clonal complexes of *S. aureus*.

Beside the presence of virulence-associated markers or resistance genes, we also tried to analyse phylogenetic relationships. As mentioned above, not a single isolate belonged to *S. argenteus*, *S. schweitzeri*, or *S*. *roterodami* [37]. All array profiles were unique, with the exception of those of ST2990 that matched previously tested human isolates and of ST3268, which was already known from previously characterised simian isolates [17,18,21,22]. A comparison to the MLST profiles in the MLST database might indicate a possible relationship of ST7689 to ST2871 and ST3463. Unfortunately, for both STs, neither host species nor any other metadata are provided. A couple of STs previously found in Rhesus macaques (ST2097, ST2098, ST2106, ST2119; [9]) are included in the PubMLST database. However, their MLST profiles are different from those of our isolates; therefore, all of them can be considered as different, separate clonal complexes, indicating that there are even more poorly known macaque lineages that deserve further study.

Although our study provides insight into the unappreciated biodiversity of *S. aureus* in macaques, it is not known which other *S. aureus* lineages might occur in Nepalese or other wild primates. There are no quantitative data on their prevalence and no data on the natural geographic range of these *S. aureus* lineages. It would also be interesting to see if primates share *S. aureus* with other wildlife or livestock that exist in the same environment. Nepal alone hosts as many as 208 mammal and 867 bird species (https://ntnc.org.np/thematic-area/species; retrieved 18 September 2022), and all of them might harbour their own lineages, not only of *S. aureus*, but also of other potential pathogens, such as mycobacteria, corona, or pox viruses. A staggering lack on typing data concerning *S. aureus* from wildlife, livestock, and even on MSSA from human communities, especially in rural areas, makes it currently impossible to recognise zoonotic transmissions. Even if a widespread or pandemic MRSA lineage (such as CC772 or CC1153) had recently emerged from such a transmission, we would currently not be able to realise that due to a lack of knowledge of the natural history of their susceptible precursors. This emphasises the need for the study of potential pathogens in wildlife in order to detect and possibly pre-empt transmissions of such. It might be interesting to search macaques (and other wildlife) for further *S. aureus* lineages that might have emerged in the natural range of these animals and then spilled over into humans. This includes *S. aureus* CC772, CC913, CC1153, and ST2990, as these lineages appeared to be related or even identical (ST2990) to macaque strains. Conversely, a spillover of human strains into wildlife species might also be relevant regarding the protection of rare species, especially non-human primates, as discussed previously [19,20,24].

Regarding the 15 major GIs analysed, as well as to *agr* groups and capsule types, no other features indicating a possible adaption to non-human hosts could be identified beside the unusual size of the *sasG* gene in all macaque strains that actually carried it. All GI-specific markers and most combinations thereof can also be found in human strains, and data on their prevalence in monkey strains are currently unavailable.

The *sasG* gene was longer, containing a higher number of repeating units than *sasG* from human isolates usually does [43], regardless of the allelic variant actually present. Whether this was a host specific adaption needs still to be determined. Regarding a previously sequenced macaque strain (ST3268, TXA; SAMN04362246), the length of the *sasG* gene cannot be determined, as it is split across contigs. This was one reason to also sequence a ST3268 strain for the present study, which indeed was found to harbour a longer *sasG* gene than most human strains. A human ST2990 isolate might provide another clue, but unfortunately, the *sasG* sequence of a human isolate from this lineage (GenBank VCMW, [31]) cannot be analysed, being absent or split across contigs.

*S. aureus* has a high number of apparently redundant virulence factors, i.e., around twenty enterotoxins, a couple of leukocidins, etc. Some virulence factors are clearly related to host specifity. For instance, *hlb* appears to be relevant for haemolysis of ruminant erythrocytes, whereas genes located on *hlb*-converting phages (*chp*, *scn*, *sak*, *sea*; [49]) appear to be more advantageous in humans than in ruminants. Interestingly, the only monkey lineage harbouring these genes on a *hlb*-converting prophage was CC2990, which is also known from humans. However, no systematic data are available on their prevalence and pathogenetic role in *S. aureus* from non-human primates.

There are several leukocidins that clearly determine host species specificity, rendering *S. aureus* pathogenic for humans (*lukS*/*F-PV*), ruminants (*lukM*/*F-P83*), horses (*lukP*/*Q*, [47]), or beavers (*lukS*/*F-BV*; [48]), but these were all are absent from the study strains. There was also no evidence for novel leukocidin genes. Interestingly, *lukF*/*S-PV* was already found in macaque MRSA [19,20], but these strains were known to be epidemic among humans in other parts of the world, indicating that they likely were imported to Nepal from abroad. Since PVL appears not to be of pathogenic relevance in macaques [50], its detection in these animals might indicate a recent anthropozoonotic transmission of *S. aureus* lineages from humans to monkeys, which hopefully does not pose a major biological risk to possibly endangered macaque populations.

Five of the macaque-associated clonal complexes—including CC2990 (which was also found in humans [30,31,32])—harboured a pathogenicity island carrying *edinB* and *etD2*/*etE* (ST2990, ST7687, ST7690, ST7749, and ST7750). This is a remarkably high rate. For comparison, out of the other more than 100 lineages of *S. aureus*, only 4 are known to carry these genes. These include CC130, ST2616, ST2867 and ST2970. CC130 is widespread among small, wild mammals (especially hedgehogs, in which *mecC*-MRSA evolved [51,52,53,54,55,56]) and in small ruminants. ST2616 strains either originate from humans, or there are no data provided (see MLST database, sequence type query page; https://pubmlst.org/bigsdb?page=query&designation_field1=s_1_ST&designation_value1=2616&db=pubmlst_saureus_isolates&order=id&submit=1&set_id=0&designation_operator1==; accessed 18 September 2022). However, the presence of *mecC* might suggest an origin in small, wild mammals. ST2867 was found in humans from the Middle East [32] and France (SAMEA698399). ST2970 (SAMEA3448974) originated from Thailand, i.e., from the geographical range of macaques, but unfortunately no metadata are provided for that sequence. Other pathogenicity islands on which *edinB* is located can be found in common human lineages of *S. aureus*, such as CC20, CC80, or CC152. However, in human-associated lineages, *edinB* is usually associated with *etd*, another exfoliative toxin gene (e.g., in CC20 and CC80). In conclusion, the presence of the pathogenicity island comprising both *edinB* and *etD2*/*etE* might tentatively be regarded as a marker indicating the recent zoonotic transmission of an *S. aureus* lineage from animals to humans. However, epidemiological studies alone cannot fully clarify issues of host specificity, and animal experiments are clearly beyond the scope of the present study. Nevertheless, the role of *edinB* in human and non-human primate or other animal tissues might be an interesting topic for a study as well as a cross-species comparison of the effects of *etD* and *etD2*/*etE.*

The absence of antimicrobial resistance genes from the presumably native “wild monkey” lineages is conspicuous, especially given the frequent detection of “human-associated” lineages of MRSA in temple monkeys. As discussed above, we assume that Middle-Eastern strains were imported and that they were transmitted to monkeys that live in proximity to humans. However, the ST2817/ST3268 complex must be regarded as a separate issue. This MRSA strain was never described in humans, but there are several observations from unrelated settings (USA, China, Singapore; see above). To the best of our knowledge, there are also no reports on MSSA from this lineage. Whether this can be attributed to a rare occurrence of ST2817/ST3268-MSSA or to a mere lack of typing data is not yet clear. However, the almost complete identity of its SCC*mec* element to the one present in the European CC398 LA-MRSA suggests the transmission of this element and its integration into a native monkey strain. The feeding of animals colonised by such a strain with meat or offal contaminated with the CC398 LA-MRSA might thus have resulted into the emergence of ST2817/ST3268 MRSA. Theoretically, this transmission might also have occurred from ST3268 to CC398, but we assume that CC398 was more likely the source of this element because this strain is more common and widespread, and because it has been extant for a much longer time.

A limitation to this study is the small sample size, resulting from opportunistic sampling at various locations. In order to obtain more comprehensive insight into *S. aureus* populations in wild animal and/or macaque hosts, as well as into their carriage of mobile genetic elements possibly carrying virulence factors or resistance genes, many more isolates should be systematically sampled, also considering clinical presentations, and sequenced. This, however, is not easy for wildlife, given the fact that, in large parts of the world, even routine cultures for diagnostic samples from human patients are hardly affordable. Another limitation is that the study was strictly sequence based. Thus, it was impossible to determine if the expression rather than the mere presence of virulence factors might be related to host-specific traits or adaptions. This also could be a topic for future studies.

## 4. Materials and Methods

### 4.1. Sampling of Wild Primates in Nepal

Isolates were collected during previous work, and sampling procedures and sites have already been described in detail [20]. Eleven locations were sampled, which represent religious/temples sites, including Bajrayogini, Nilbarahi, Pashupati, Swayambhu, Thapathali, Chitwan, Guheswari, Gokarna, Hetauda, Rupandehi, and Ramdi. Human dwellings were re-situated a few hundred meters from the temple. The diet of these macaques consisted of food from the forest, but they also were fed with fruits and household scraps by the local people and pilgrims visiting the temples. All of the locations involved rhesus macaques, with the exception of Ramdi, where a resident group of Assam macaques was sampled. The sample collection technique was based on a non-invasive method using SalivaBio Children’s Swabs (Salimetrics LLC, State College PA, USA) [19,20,57]. Swabs were soaked in a sterile glucose solution (10% *w*/*v*) and were tossed to the macaques. After chewing for a short time, the monkeys discarded the swabs upon realising that they were not edible. The swabs were then collected and placed into a tube containing enrichment broth (Bacto-m-Staphylococcus Broth^®^; Difco Laboratories, Sparks, MD, USA; supplemented with 75 mg/L of polymyxin B, 0.01% potassium tellurite and either with or without 12.5 mg/L nystatin; Sigma-Aldrich, St Louis, MO, USA). Tubes were returned to the laboratory, where an aliquot of the broth was spread on Colombia blood agar.

Bacterial colonies that showed beta-haemolysis on blood agar plates were verified as *S. aureus* by Gram stain and with the Staphaurex test (Thermo Fisher Scientific Remel Products, Lenexa, KS, USA). Isolates were characterised by a microarray-based assay [20,46]. For the present study, 18 isolates were selected, which yielded previously unseen microarray hybridisation patterns and/or novel MLST alleles. In addition, an ST2990 isolate was included, representing a lineage that was not entirely new but that was still only rarely described.

### 4.2. Sampling of ST3268

This was an isolate from a Rhesus macaque held in the Washington National Primate Research Center (WaNPRC), and it was obtained during surveillance after an outbreak investigation [18,21]. Details have been described in a previous paper (where it was referred to as A140, [18]).

### 4.3. Sampling of Primates in a German Zoo

Isolates were collected at Erfurt Zoopark, Thuringia, Germany, which has an enclosure of approximately one hectare where, at the time of investigation, a group of 28 Barbary macaques were living semi-free. This area is a walkable free enclosure for visitors, and it is shaped close to nature. Feeding by visitors is strictly prohibited. For the cultivation of staphylococci, faecal samples and nasal swabs were used. The latter were obtained from twelve macaques, which were captured in order to be transferred to other zoos. Other animals were not sampled to avoid any stress.

Swabs and 1 g of fecal samples were added to 10 mL of Mueller–Hinton broth (Oxoid GmbH, Wesel, Germany) with 6% NaCl and were incubated at 37 °C for 24 h. Aliquots of these cultures were streaked on Baird-Parker agar (Sifin Diagnostics GmbH, Berlin, Germany), and plates were incubated at 37 °C for 24 h. Black colonies suspicious as staphylococci were sub-cultured on blood agar at 37 °C for 24 h. Isolates were identified using conventional standard procedures for *S. aureus* identification and MALDI-ToF mass spectrometry.

Five isolates were thus identified as *S. aureus*, and others belonged—according to MALDI-ToF—to *S. arlettae*, *S. chromogenes*, *S. equorum*, *S. haemolyticus*, *S. simulans*, *S. succinus*, and *S. warneri*, as well as *Mammaliicoccus* (M.) *fleuretti* and *M. sciuri*. All *S. aureus* isolates were typed using a DNA microarray [46]. Two *S. aureus* strains from two different animals were assigned to CC49. This is an animal-associated lineage known also from various wild European rodents [48,58] and livestock [59], for which genome sequences are already available [48,60]. Thus, these isolates were not sequenced and are not discussed herein. Two isolates yielded identical but unknown hybridisation patterns, one of which (16CS0212) was further characterised. A fifth isolate (16CS0209) presented with yet another unknown pattern on the array and was also sequenced for the present study.

### 4.4. Array Experiments

All isolates were, prior to sequencing, characterised using microarrays, and the decision to sequence was made based on their rare or unknown hybridisation patterns. The arrays, protocols, and procedures, as well as the probe sequences, have been described previously in detail [18,20,46,61].

### 4.5. Illumina Sequencing

Both Erfurt strains and 17 of the Nepalese strains were subjected to whole-genome sequencing (WGS) using Illumina technology. DNA was extracted using the QIAamp^®^ DNA Mini Kit (QIAGEN, Hilden, Germany) with a protocol adapted for staphylococci, as described previously [62]. The WGS libraries were prepared using the Nextera XT DNA Library Preparation Kit (Illumina, Inc., San Diego, CA, USA) following the manufacturer’s recommendations. The 2 × 300 bp paired-end sequencing in 40-fold multiplexes was performed on the Illumina MiSeq platform (Illumina, Inc., San Diego, CA, USA).

### 4.6. Nanopore Sequencing

The Oxford Nanopore MinION platform was used for the WGS of all monkey isolates. Genomic DNA was isolated from an overnight culture grown at 37 °C on Columbia Blood Agar plates (Becton Dickinson GmbH, Heidelberg, Germany) using a Macherey and Nagel NucleoSpin^®^ Microbial DNA kit (MACHEREY-NAGEL GmbH & Co. KG, Dueren, Germany). Briefly, size selection and DNA clean-up were performed using Agencourt AMPure XP beads (Beckman Coulter GmbH, Krefeld, Germany) in a ratio of 1/1 (*v*/*v*). The DNA library was generated using the Nanopore native barcoding genomic DNA kit SQK-LSK109 in combination with the native barcoding expansion kit EXP-NBD104 (Oxford Nanopore Technologies, Oxford, UK) according to the manufacturer’s instructions. The used flow cell FLO-MIN106 (revD R9.4.1) was primed by the flow cell priming kit EXP-FLP001 (Oxford Nanopore, Oxford, UK). The protocol named “Native barcoding genomic DNA” was used in version NBE_9065_v109_revV_14Aug2019 (last update: 21 February 2020).

The Guppy basecaller (version 5.0.16 up to 6.0.6+8a98bbcbd, depending on the time of the sequencing of the actual strain, Oxford Nanopore Technologies, Oxford, UK) translated the MinION raw reads (FAST5) into quality tagged sequence reads (4000 reads per FASTQ-file) using the barcode trimming option. Flye (v2.8.3-b1695) was used to assemble the quality tagged sequence reads of each strain to one big circular contig (for length and coverage, see Table 1). The polishing of assemblies was divided into two steps. First, racon (v1.4.21) was iteratively used four times with the following parameters: match 8; mismatch 6; gap 8; and window length 500. Afterwards, medaka (v1.4.3) ran on the last racon polished assembly using the model r941_min_high_g360. Finally, Pylon (v1.23) was used to polish nanopore sequences using the Illumina data for those strains, for which they were available. Corrected assemblies were used for further analysis.

### 4.7. Core Genome Analysis

We selected 154 core genomic markers for phylogenetic analysis, as previously discussed [37,39]. The inclusion criteria were their presence in all *S. aureus*/*argenteus*/*schweitzeri*/*roterodami* clonal complexes and their uniform length in all published genomes. A total of 157 genomes were analysed. For the sake of simplicity, genomes of strains that were known to be chimeras or hybrids, consisting of fragments originating from unrelated parental lineages, were excluded (ST34, ST71, variant ST80, ST239, ST567, ST2249, and ST6610; [63,64,65,66,67]). Genes and strains are listed in Supplemental File S3. Sequences were concatenated and analysed using SplitsTree 4.0 [36] on default settings (characters transformation, uncorrected P; distance transformation, Neighbour-Net; and variance, ordinary least squares).

## 5. Conclusions

Twenty-two complete genome sequences of novel or poorly known *S. aureus* strains from three species of macaques were obtained by nanopore and Illumina sequencing and were analysed. This allowed for the definition of twenty novel MLST sequence types. Although our study provides insight into the unappreciated biodiversity of *S. aureus* in macaques, it is not known which other *S. aureus* lineages might occur in Nepalese or other wild primates. Further studies are necessary to assess the risk of possible spill-over of zoonotic *S. aureus* lineages into humans, and based on the phylogenetic analysis of the study strains, human strains of CC772, CC1153, and CC2990 could have emerged in this way. There was no evidence for novel host-specific virulence factors. However, a conspicuously high rate of carriage of the pathogenicity island harbouring *edinB* and *etD2*/*etE*, as well as a higher number of repeat units within the gene *sasG* than in human isolates, were observed. Although antimicrobial resistance genes were rare, one of the study strains (ST3268) harboured an SCC*mec* VT element. This proved to be virtually identical to the one from the known livestock-associated CC398-MRSA strain, suggesting this strain to be a source for the acquisition of that element.

## Figures and Tables

**Table 1:** Origin and typing data of study strains.

Sequence Type	Isolate ID	GenBank Accession No.	Ref.	Origin	Host	Genome Length (nt)	Coverage (Nanopore)	MLST Profile (arcC-aroE-glpF-gmk-pta-tpi-yqiL)	agr Group	Capsule Type
ST2990	27-G-H	CP102977	[20]	Guheswori, Nepal, 2019	Rhesus macaque	2,734,401	249	1-1-1-1-330-1-10	II	8
ST3268	Ma2/A14043	CP102976	[18]	USA, 2015	Rhesus macaque	2,858,627	646	1-14-430-214-10-303-329	IV	5
ST4168	16CS0209	CP102975	N/A	Erfurt, Germany, 2016	Barbary macaque	2,769,978	181	102-176-6-2-6-440-419	III	5
ST7687	01-RR-86	CP102974	[20]	Ralpa-Ramdi, Nepal, 2019	Assam macaque	2,761,089	143	4-13-1-105-11-5-850	I	5
ST7688	05-RR-90	CP102972-973	[20]	Ralpa-Ramdi, Nepal, 2019	Assam macaque	2,822,476	189	3-1-1-66-28-1-850	III	5
ST7689	08-G-E	CP102971	[20]	Gokarna, Nepal, 2019	Rhesus macaque	2,752,591	85	1-421-1-1-12-1-11	IV	5
ST7690	09-G-F	CP102970	[20]	Gokarna, Nepal, 2019	Rhesus macaque	2,815,864	102	1-1-1-1-28-4-11	I	8
ST7691	13-G-52	CP102968-969	[20]	Gokarna, Nepal, 2019	Rhesus macaque	2,738,477	136	1-421-1-1-12-238-11	I	5
ST7692	17-H-61	CP102967	[20]	Hetauda, Nepal, 2019	Rhesus macaque	2,748,100	192	4-421-1-105-1-5-854	IV	5
ST7693	29-P-01	CP102966	[20]	Pashupati, Nepal, 2019	Rhesus macaque	2,771,670	52	100-1-1-15-1-4-11	I	8
ST7694	40-B-50	CP102963-965	[20]	Bajrayogini, Nepal, 2019	Rhesus macaque	2,818,795	252	100-1-1-1-28-1-11	III	8
ST7695	16CS0212	CP102962	N/A	Erfurt, Germany, 2016	Barbary macaque	2,822,504	164	6-79-12-2-13-50-172	I	8
ST7745	03-RR-88	CP102961	[20]	Ralpa-Ramdi, Nepal, 2019	Assam macaque	2,800,466	201	1-38-1-1-1-238-1013 *	II	8
ST7746	07-G-D	CP102960	[20]	Gokarna, Nepal, 2019	Rhesus macaque	2,773,903	51	1-3-1-15-28-840-1	I	8
ST7747	12-G-51	CP102959	[20]	Gokarna, Nepal, 2019	Rhesus macaque	2,743,617	197	12-1087-1-66-11-839-850	II	8
ST7748	15-G-54	CP102958	[20]	Gokarna, Nepal, 2019	Rhesus macaque	2,715,191	161	1-421-1-598-916-1-11	I	8
ST7749	18-H-62	CP102957	[20]	Hetauda, Nepal, 2019	Rhesus macaque	2,719,389	109	1-3-1-598-1-1-11	IV	5
ST7750	26-G-G	CP102956	[20]	Guheswori, Nepal, 2019	Rhesus macaque	2,791,132	138	3-38-1-15-1-841-40	IV	5
ST7751	28-G-I	CP102955	[20]	Guheswori, Nepal, 2019	Rhesus macaque	2,749,231	128	3-3-1-66-4-1-1014	IV	8
ST7752	30-P-10	CP102954	[20]	Pashupati, Nepal, 2019	Rhesus macaque	2,787,121	129	3-3-1-66-28-842-850	II	8
ST7753	32-T-13	CP102953	[20]	Thapthali, Nepal, 2019	Rhesus macaque	2,773,149	167	4-3-1-598-12-1-11	IV	8
ST7754	39-B-49	CP102952	[20]	Bajrayogini, Nepal, 2019	Rhesus macaque	2,760,481	258	3-1088-943-105-12-10-13	IV	8

* Based on the Nanopore sequence. Previous conventional sequencing of MLST PCR products yielded *yqiL*-852, which differs in one nucleotide.

**Table 3:** The pathogenicity island carrying *edinB* and *etD2*/*etE* (see also Figure 3).

Gene ID	Description	Length	Direction	Coordinates in the Reference Sequence, CC130 Strain O11, CP024649.1	Locus Tag in the Reference Sequence, CC130 Strain O11
Q5HE01	peptidase, M23/M37 domain family	855	Reverse	(2,247,871 to 2,248,725)	SaO11_02006
*hsdS_etd*	type I restriction–modification system site-specificity determinate associated with *etD* and *etD2*/*etE*	1221	Reverse	(2,248,976 to 2,250,196)	SaO11_02007
*hsdM*	type I restriction–modification system DNA methylase	1557	Reverse	(2,250,189 to 2,251,748)	SaO11_02008
F3TKB7	glutamyl-endopeptidase	699	Forward	(2,252,060 to 2,252,758)	SaO11_02009
*edinB*	epidermal cell differentiation inhibitor precursor	756	Forward	(2,252,804 to 2,253,547)	SaO11_02010
*etD2*/*etE*	exfoliative toxin D2 or E	842	Reverse	(2,253,775 to 2,254,616)	SaO11_02011
*istB2*	transposase IS712G helper protein	768	Reverse	N/A; ST7690 (09-G-F) only	N/A
*tnp*_IS712G	transposase for IS712G	1235	Reverse	N/A; ST7690 (09-G-F) only	N/A
F0D4L5	putative DNA helicase	1086	Reverse	(2,254,979 to 2,256,064)	SaO11_02012
F0D4L4	putative DNA binding protein	1545	Reverse	(2,256,049 to 2,257,593)	SaO11_02013
Q5HE00	HAD-superfamily hydrolase	816	Reverse	(2,257,721 to 2,258,536)	SaO11_02014

**Table 4:** Prophages and their integration sites.

Sequence Type (Isolate ID)	Between *trfA* and *trfB*, around Pos. 950,000	Between *per* and *psmB1*, around Pos. 1,100,000	Between *glnA* and A6U1C8, around Pos. 1,300,000	Between Q2YXQ4 and A6QGL8, around Pos. 1,300,000	Within A5IT17, around Pos. 1,550,000	Within A5IU43 = *yfkAB*, around Pos. 1,950,000	Within *hlb*, around Pos. 1,970,000	Between *alsD*-L1 and *rpsI*, around Pos. 2,200,000	Between *rsr* and *iraC*, Replacing *iraD*, around Pos. 2,300,000
ST2990 (27-G-H)	-	-	-	Fragment	-	-	Sipho, carrying *sak*/*chp*/*scn*	-	-
ST3268 (Ma2/A14043)	-	-	Unident./fragm.	Fragment	Sipho	-	-	-	-
ST4168 (16CS0209)	-	-	Unident./fragm.	Fragment	-	-	-	-	-
ST7687 (01-RR-86)	-	-	-	Fragment	-	-	-	-	-
ST7688 (05-RR-90)	-	-	-	-	-	Sipho	-	-	Sipho
ST7689 (08-G-E)	-	-	Unident./fragm.	-	-	-	-	-	-
ST7690 (09-G-F)	-	-	Unident./fragm.	-	-	-	-	-	Sipho
ST7691 (13-G-52)	-	-	-	Fragment	-	-	-	-	-
ST7692 (17-H-61)	Sipho	-	-	Fragment	-	-	-	-	-
ST7693 (29-P-01)	-	Sipho	-	Fragment	-	-	-	-	-
ST7694 (40-B-50)	-	Sipho	Unident./fragm.	Fragment	-	-	-	-	Sipho
ST7695 (16CS0212)	-	Sipho	Unident./fragm.	Fragment	-	-	-	-	-
ST7745 (03-RR-88)	-	-	Unident./fragm.	Fragment	Sipho	-	-	-	-
ST7746 (07-G-D)	-	-	-	-	-	-	-	-	Sipho
ST7747 (12-G-51)	-	-	-	-	-	-	-	-	-
ST7748 (15-G-54)	-	-	-	Fragment	-	-	-	-	-
ST7749 (18-H-62)	-	-	-	-	-	-	-	-	-
ST7750 (26-G-G)	-	-	-	Fragment	-	-	-	-	-
ST7751 (28-G-I)	-	-	Unident./fragm.	-	-	-	-	-	-
ST7752 (30-P-10)	-	-	-	-	-	-	-	-	Sipho
ST7753 (32-T-13)	-	-	-	Fragment	-	-	-	-	Sipho
ST7754 (39-B-49)	-	-	-	Fragment	-	-	-	-	Sipho

**Table 5:** The SCC*mec* element in ST3268-MRSA-VT compared to the one in the European CC398 LA-MRSA strain.

Gene ID	Gene Product/Description	Orientation	Start Pos. in ST3268	End Pos. in ST3268	Length in ST3268	Start Pos. in AM990992	End Pos. in AM990992	Length in AM990992	Locus Tag in AM990992
*orfX*	23S rRNA methyltransferase	Forward	36,094	36,571	478	33,806	34,285	480	SAPIG0027
DR-SCC	direct repeat of SCC		36,553	36,571	19	34,267	34,285	19	N/A
sccterm02	terminus of SCC towards *orfX*		36,572	36,888	317	34,286	34,602	317	N/A
Q2FKL3	HNH endonuclease family protein	Truncated	36,889	37,255	367	34,603	34,970	368	SAPIG0028
D1GU38	putative protein	Forward	37,320	38,183	864	35,035	35,898	864	SAPIG0029
D2N370	putative protein	Forward	38,291	39,766	1476	36,006	37,481	1476	SAPIG0030
Q4LAG3	putative protein	Forward	39,992	41,092	1101	37,706	38,806	1101	SAPIG0031
Q3T2M7	putative protein	Forward	41,085	41,456	372	38,799	39170	372	SAPIG0032
*ccrAA*	cassette chromosome recombinase homologue, associated with *ccrC*	Forward	41,453	43,096	1644	39,167	40,810	1644	SAPIG0033
*ccrC*	cassette chromosome recombinase C	Forward	43,322	44,998	1677	41,036	42,712	1677	SAPIG0035
Q93IE0	putative protein	Forward	45,104	45,443	340	42,818	43,156	339	N/A
Q0P7G0	putative protein	Forward	45,539	45,850	312	43,252	43,563	312	SAPIG0036
Q9KX75	putative protein	Forward	45,866	46,372	507	43,579	44,085	507	SAPIG0037
IR_IS*431*	inverted repeat of IS*431*	-	46,462	46,477	16	44,175	44,190	16	N/A
*tnp*_IS*431*	transposase for IS*431*	Reverse	46,521	47,195	675	44,234	44,908	675	SAPIG0038
Teg143	trans-encoded RNA associated with tnpIS*431*	-	47,226	47,259	34	44,939	44,972	34	N/A
IR_IS431	inverted repeat of IS*431*	-	47,236	47,251	16	44,949	44,964	16	N/A
*mvaS*-SCC	truncated 3-hydroxy-3-methylglutaryl CoA synthase	Forward (frameshift)	47,268	47,620	353	44,981	45,333	353	SAPIG0039
Q5HJW6	putative protein	Forward	47,718	47,948	231	45,431	45,661	231	N/A
*dru*	SCC direct repeat units	-	47,858	48,335	478	45,571	46,008	438	N/A
*ugpQ*	glycerophosphoryl diester phosphodiesterase	Forward	48,537	49,280	744	46,210	46,953	744	SAPIG0040
*ydeM*	putative dehydratase	Forward	49,377	49,805	429	47,050	47,478	429	SAPIG0041
*txbi_mecA*	bidirectional rho-independent terminator of *mecA*	-	49,796	49,860	65	47,469	47,533	65	N/A
*mecA*	penicillin-binding protein 2a	Reverse	49,851	51,857	2007	47,524	49,530	2007	SAPIG0042
*mecR1*_trunc. *(mec* complex C)	methicillin resistance operon repressor, truncated in SCC*mec* V	Forward/truncated	51,957	51,973	17	49,630	49,646	17	N/A
IR_IS*431*	inverted repeat of IS*431*	-	52,049	52,064	16	49,722	49,737	16	N/A
*tnp*_IS*431*	transposase for IS*431*	Forward	52,105	52,779	675	49,778	50,452	675	SAPIG0043
Q4LAG7	putative protein	Reverse	52,839	53,267	429	50,512	50,940	429	SAPIG0044
*yobV*	transcriptional regulator	Forward	53,348	54,277	930	51,021	51,950	930	SAPIG0045
Q4LAG4	putative protein	Forward	54,439	56,427	1989	52,112	54,100	1989	SAPIG0046
Q4LAG3	putative protein	Forward	56,622	57,731	1110	54,295	55,404	1110	SAPIG0047
Q3T2M7	putative protein	Forward	57,724	58,092	369	55,397	55,765	369	SAPIG0048
*ccrAA*	cassette chromosome recombinase homologue associated with *ccrC*	Forward	58,092	59,708	1617	55,765	57,381	1617	SAPIG0049
*ccrC*	cassette chromosome recombinase C	Forward	59,933	61,612	1680	57,606	59,285	1680	SAPIG0050
Q4LAF9	putative protein	Forward	61,701	62,038	338	59,374	59,712	339	SAPIG0051
Q7A206-delta	putative protein	Forward/truncated	62,044	62,130	87	59,718	59,804	87	N/A
Q7A207	putative protein	Forward	62,132	62,443	312	59,806	60,117	312	SAPIG0052
Q9KX75	putative protein	Forward	62,449	62,928	480	60,123	60,602	480	SAPIG0053
IR_IS*431*	inverted repeat of IS*431*	-	62,911	62,926	16	60,585	60,600	16	N/A
*tnp*_IS*431*	transposase for IS*431*	Reverse	62,969	63,643	675	60,643	61,317	675	SAPIG0054
IR_IS*431*	inverted repeat of IS*431*	-	63,684	63,699	16	61,358	61,373	16	N/A
*tet*(K)	tetracycline efflux protein variant K	Forward	63,840	65,219	1380	N/A	N/A	N/A	N/A
*pre4*_pT181	plasmid replication protein	Forward	65,405	66,646	1242	N/A	N/A	N/A	N/A
ctRNA_pT181	counter-transcribed RNA	-	66,984	67,078	95	N/A	N/A	N/A	N/A
*tnp*_IS*1*	transposase for IS1	Reverse	67,188	68,174	987	N/A	N/A	N/A	N/A
*repD*_pT181	plasmid replication initiation protein	Forward	68,408	69,201	794	N/A	N/A	N/A	N/A
IR_IS*431*	inverted repeat of IS*431*	-	69,231	69,246	16	N/A	N/A	N/A	N/A
*tnp*_IS*431*	transposase for IS*431*	Reverse	69,289	69,963	675	N/A	N/A	N/A	N/A
IR_IS*431*	inverted repeat of IS*431*	-	70,004	70,019	16	N/A	N/A	N/A	N/A
*top3d (topB)*	DNA topoisomerase III type IA	Forward	70,109	70,636	528	61,475	62,002	528	SAPIG0055
*cch*	cassette chromosome helicase	Truncated	70,935	71,549	615	62,301	62,915	615	SAPIG0056
D2N398	putative protein	Reverse	72,001	72,360	360	63,367	63,726	360	SAPIG0058
*yozA*	HTH-type transcriptional repressor	Forward	72,567	72,893	327	63,933	64,259	327	N/A
*czrC*	cadmium and zinc resistance gene C	Forward	73,214	75,148	1935	64,580	66,514	1935	SAPIG0059
*cstB*-SCC	*CsoR*-like sulfur transferase-regulated gene B	Reverse	76,293	77,621	1329	67,660	68,985	1326	SAPIG0061
*cstA*-SCC	*CsoR*-like sulfur transferase-regulated gene A	Reverse	77,640	78,707	1068	69,004	70,071	1068	SAPIG0062
*cstR*-SCC	copper-sensing transcriptional repressor	Forward	78,846	79,102	257	70,210	70,466	257	SAPIG0063
DUF81-SCC	putative sulfite/sulfonate efflux	Forward	79,130	79,861	732	70,494	71,225	732	SAPIG0064
*copA2*-SCC	copper-exporting ATPase	Forward/truncated	80,031	80,240	210	71,395	71,604	210	SAPIG0065
*ydhK*	putative lipoprotein	Forward	80,258	80,803	546	71,622	72,167	546	SAPIG0066
DR-SCC	direct repeat of SCC	-	81,008	81,026	19	72,372	72,390	19	N/A
D2N3A7	putative protein	Forward	81,084	82,875	1792	72,448	74,238	1791	SAPIG0067
F8WKF7	putative protein	Forward, truncated in AM990992	82,916	84,073	1158	74,279	74,897	619	SAPIG0068

## Data Availability

All genome sequences discussed can be retrieved via GenBank accession numbers CP 102952 to CP 102977, BioProject Accession No.: PRJNA870416, and are additionally provided as Supplemental Files.

## Supplementary Materials

The following supporting information can be downloaded at: https://www.mdpi.com/article/10.3390/ijms231911225/s1.
