# Supplementary material for: Sequence Analysis of Novel Staphylococcus aureus Lineages from Wild and Captive Macaques
Source: Int J Mol Sci. 2022 Sep 23;23(19):11225. doi: 10.3390/ijms231911225 (PMC9570271; doi:10.3390/ijms231911225)
Supplement: Supplementary file 1 [file ijms-23-11225-s001.zip › Supplemental File S3a_Genome sequences and target genes used for the construction of the SplitsTree graph.pdf]

1. Genes used for tree construction

Gene ID	GenBank coordinates	ID used in PubMLST cgMLST scheme
**dnaA**	(MW2-BA000033-[517:1878])	SAUR0001 (SAR_RS00005)
**gyrB**	(MW2-BA000033-[5034:6968])	SAUR0005 (SAR_RS00025)
**walR**	(MW2-BA000033-[24930:25631])	SAUR0020 (SAR_RS00100)
**walK**	(MW2-BA000033-[25644:27470])	SAUR0021 (SAR_RS00105)
**orfX**	(MW2-BA000033-[33689:34168])	SAUR0026 (SAR_RS00130)
**sarS**	(CA347-CP006044-[136199:136951])	
**sirB**	(MW2-BA000033-[103544:104539])	SAUR0111 (SAR_RS00555)
**sbnB**	(MW2-BA000033-[106755:107765])	SAUR0114 (SAR_RS00570)
**sbnI**	(MW2-BA000033-[116231:116995])	SAUR0121 (SAR_RS00605)
**rpiRB**	(MW2-BA000033-[199250:200128])	
**ssaA**	(MW2-BA000033-[304578:305480])	
**esaB**	(N315-BA000018-[330908:331150])	SAUR0284 (SAR_RS01420)
**thlA=yqil**	(MW2-BA000033-[378859:380040])	SAUR0353 (SAR_RS01765)
**rpsR**	(MW2-BA000033-[392495:392737])	
**nfrA**	(MW2-BA000033-[404682:405437])	SAUR0403 (SAR_RS02015)
**xpt**	(MW2-BA000033-[410879:411457])	SAUR0409 (SAR_RS02045)
**pbuX**	(MW2-BA000033-[411457:412725])	SAUR0410 (SAR_RS02050)
**mpsC=ybcI**	(MW2-BA000033-[460582:460944])	SAUR0450 (SAR_RS02250)
**yaaK**	(N315-BA000018-[504381:504698])	SAUR0477 (SAR_RS02385)
**recR**	(MW2-BA000033-[490084:490680])	SAUR0478 (SAR_RS02390)
**glmU**	(MW2-BA000033-[512249:513601])	SAUR0505 (SAR_RS02525)
**pth**	(MW2-BA000033-[515827:516399])	SAUR0509 (SAR_RS02545)
**ctsR**	(N315-BA000018-[560629:561090])	SAUR0546 (SAR_RS02730)
**mrnC**	(N315-BA000018-[572547:572951])	
**yacO**	(MW2-BA000033-[563521:564267])	SAUR0557 (SAR_RS02785)
**secE**	(COL-CP000046-[602561:602743])	
**rplK**	(N315-BA000018-[576117:576539])	SAUR0562 (SAR_RS02810)
**rplA**	(11819-97-CP003194-[574818:575510])	
**rpoC**	(MW2-BA000033-[573870:577493])	SAUR0568 (SAR_RS02840)
**tuf**	(MW2-BA000033-[581352:582536])	SAUR0573 (SAR_RS02865)
**thiD1**	(11819-97-CP003194-[629476:630306])	SAUR0603 (SAR_RS03015)
**pta**	(11819-97-CP003194-[636472:637458])	SAUR0611 (SAR_RS03055)
**mvaD**	(MW2-BA000033-[632126:633109])	SAUR0614 (SAR_RS03070)
**mvaK2**	(MW2-BA000033-[633122:634198])	SAUR0615 (SAR_RS03075)
**A5IQH4**	(CA347-CP006044-[681325:681885])	
**mrpE**	(MW2-BA000033-[671224:671706])	SAUR0651 (SAR_RS03255)
**mntB**	(N315-BA000018-[681665:682501])	SAUR0659 (SAR_RS03295)
**mntA**	(CA347-CP006044-[692970:693713])	
**mntR**	(MW2-BA000033-[678714:679358])	SAUR0661 (SAR_RS03305)
**tarH**	(MW2-BA000033-[681251:682045])	SAUR0664 (SAR_RS03320)
**yxkD**	(MW2-BA000033-[691596:692429])	SAUR0673 (SAR_RS03365)
**dakL**	(MW2-BA000033-[696806:697390])	SAUR0678 (SAR_RS03390)
**murB**	(N315-BA000018-[791508:792431])	SAUR0806 (SAR_RS04030)
**gapA**	(MW2-BA000033-[827901:828911])	SAUR0845 (SAR_RS04225)
**tpi**	(MW2-BA000033-[830362:831123])	SAUR0847 (SAR_RS04235)

Gene ID	GenBank coordinates	ID used in PubMLST cgMLST scheme
**secG**	(CA347-CP006044-[851386:851619])	
**est**	(MW2-BA000033-[835290:836030])	SAUR0852 (SAR_RS04260)
**ssrP**	(MW2-BA000033-[838458:838922])	SAUR0854 (SAR_RS04270)
**cspC-L1**	(MSHR1132-FR821777-[843125:843325])	
**ntrA**	(MW2-BA000033-[872472:873011])	SAUR0878 (SAR_RS04390)
**metN1**	(MW2-BA000033-[877242:878267])	SAUR0886 (SAR_RS04430)
**dltD**	(N315-BA000018-[901367:902542])	SAUR0914 (SAR_RS04570)
**nfuA**	(MW2-BA000033-[902717:902938])	
**kapB**	(MW2-BA000033-[918173:918556])	SAUR0932 (SAR_RS04660)
**pgi**	(MW2-BA000033-[928831:930162])	SAUR0941 (SAR_RS04705)
**sufT=paaD**	(MW2-BA000033-[943736:944044])	SAUR0952 (SAR_RS04760)
**A6U088**	(N315-BA000018-[954670:954855])	SAUR0961 (SAR_RS04805)
**fabH**	(MW2-BA000033-[955118:956059])	SAUR0962 (SAR_RS04810)
**ppnK**	(MW2-BA000033-[978805:979614])	SAUR0990 (SAR_RS04950)
**ugtP**	(MW2-BA000033-[990850:992025])	SAUR1002 (SAR_RS05010)
**defB=def1**	(MW2-BA000033-[1070556:1071107])	SAUR1086 (SAR_RS05430)
**pdhD**	(MW2-BA000033-[1075749:1077155])	SAUR1091 (SAR_RS05455)
**potD**	(MW2-BA000033-[1080993:1082066])	SAUR1097 (SAR_RS05485)
**ylbN=Q4L5E0**	(11819-97-CP003194-[1137445:1138002])	SAUR1122 (SAR_RS05610)
**isdG**	(MW2-BA000033-[1113916:1114239])	SAUR1132 (SAR_RS05660)
**spoU**	(11819-97-CP003194-[1146950:1147690])	SAUR1134 (SAR_RS05670)
**mraW=rsmH**	(MW2-BA000033-[1156518:1157432])	
**gmk**	(MW2-BA000033-[1190834:1191457])	SAUR1210 (SAR_RS06050)
**rlmN**	(11819-97-CP003194-[1232662:1233756])	SAUR1219 (SAR_RS06095)
**prkC=pknB**	(MW2-BA000033-[1202183:1204177])	SAUR1221 (SAR_RS06105)
**rpmB**	(RF122-AJ938182-[1174752:1174940])	
**yloU**	(11819-97-CP003194-[1239905:1240279])	SAUR1226 (SAR_RS06130)
**ftsY**	(MW2-BA000033-[1220509:1221759])	SAUR1237 (SAR_RS06185)
**ylxM**	(MW2-BA000033-[1221746:1222078])	SAUR1238 (SAR_RS06190)
**rpsP**	(N315-BA000018-[1224108:1224383])	SAUR1240 (SAR_RS06200)
**tsf**	(MW2-BA000033-[1246279:1247160])	SAUR1262 (SAR_RS06310)
**pyrH**	(MW2-BA000033-[1247297:1248019])	SAUR1263 (SAR_RS06315)
**cdsA**	(MW2-BA000033-[1249742:1250524])	SAUR1266 (SAR_RS06330)
**rbfA**	(N315-BA000018-[1263268:1263618])	SAUR1275 (SAR_RS06375)
**ribF**	(MW2-BA000033-[1264851:1265822])	SAUR1277 (SAR_RS06385)
**rpsO**	(N315-BA000018-[1265805:1266074])	SAUR1278 (SAR_RS06390)
**pgsA**	(N315-BA000018-[1278452:1279030])	SAUR1288 (SAR_RS06440)
**glpF**	(N315-BA000018-[1296691:1297509])	SAUR1303 (SAR_RS06515)
**glpK**	(MW2-BA000033-[1297775:1299271])	SAUR1304 (SAR_RS06520)
**Q99UF3**	(N315-BA000018-[1321100:1321288])	SAUR1359 (SAR_RS06795)
**alsT**	(MW2-BA000033-[1360967:1362427])	SAUR1397 (SAR_RS06985)
**msrA1**	(MW2-BA000033-[1368327:1368836])	SAUR1402 (SAR_RS07010)
**tyrA**	(MW2-BA000033-[1371775:1372866])	SAUR1407 (SAR_RS07035)
**dapD**	(MW2-BA000033-[1405382:1406101])	SAUR1438 (SAR_RS07190)
**cspC-L2=msaB**	(CA347-CP006044-[1416364:1416564])	
**asnS**	(MW2-BA000033-[1493287:1494579])	SAUR1498 (SAR_RS07490)
**ypiA**	(MW2-BA000033-[1503738:1504982])	SAUR1507 (SAR_RS07535)

Gene ID	GenBank coordinates	ID used in PubMLST cgMLST scheme
**ndk**	(MW2-BA000033-[1509350:1509799])	SAUR1511 (SAR_RS07555)
**ubiE**	(MW2-BA000033-[1510852:1511577])	SAUR1513 (SAR_RS07565)
**ansA**	(MW2-BA000033-[1518182:1519150])	SAUR1524 (SAR_RS07620)
**xerD**	(N315-BA000018-[1536101:1536988])	SAUR1611 (SAR_RS08055)
**lpdA**	(MW2-BA000033-[1604342:1605763])	SAUR1635 (SAR_RS08175)
**xseA**	(MW2-BA000033-[1609575:1610912])	SAUR1640 (SAR_RS08200)
**efp**	(MW2-BA000033-[1614048:1614605])	SAUR1645 (SAR_RS08225)
**glk**	(N315-BA000018-[1582174:1583160])	SAUR1664 (SAR_RS08320)
**sodA-L2**	(MW2-BA000033-[1633614:1634213])	SAUR1670 (SAR_RS08350)
**dgkA**	(N315-BA000018-[1604006:1604350])	SAUR1686 (SAR_RS08430)
**prmA**	(MW2-BA000033-[1656890:1657828])	SAUR1695 (SAR_RS08475)
**dnaK**	(11819-97-CP003194-[1688529:1690361])	SAUR1697 (SAR_RS08485)
**aroE**	(MW2-BA000033-[1673986:1674792])	SAUR1712 (SAR_RS08560)
**hisS**	(CA347-CP006044-[1711663:1712925])	
**yajC**	(MRSA252-BX571856-[1784283:1784543])	
**ruvA**	(11819-97-CP003194-[1751933:1752535])	SAUR1760 (SAR_RS08800)
**pheB**	(11819-97-CP003194-[1752549:1753007])	
**rpmA**	(11819-97-CP003194-[1755197:1755481])	SAUR1763 (SAR_RS08815)
**DUF464**	(MW2-BA000033-[1725961:1726281])	SAUR1764 (SAR_RS08820)
**rplT**	(N315-BA000018-[1710716:1711072])	SAUR1798 (SAR_RS08990)
**rpmI**	(N315-BA000018-[1711119:1711319])	SAUR1799 (SAR_RS08995)
**infC**	(MW2-BA000033-[1750369:1750896])	SAUR1800 (SAR_RS09000)
**pykA**	(MW2-BA000033-[1773276:1775033])	SAUR1818 (SAR_RS09090)
**pfkA**	(MW2-BA000033-[1775055:1776023])	SAUR1819 (SAR_RS09095)
**accA**	(N315-BA000018-[1737273:1738217])	SAUR1820 (SAR_RS09100)
**tpx**	(MW2-BA000033-[1793401:1793895])	SAUR1834 (SAR_RS09170)
**yoxC**	(N315-BA000018-[1788940:1789431])	SAUR1866 (SAR_RS09330)
**pepA1**	(MW2-BA000033-[1834312:1835388])	SAUR1872 (SAR_RS09360)
**putA**	(MW2-BA000033-[1861651:1862652])	SAUR1898 (SAR_RS09490)
**menC**	(MW2-BA000033-[1884490:1885491])	SAUR1924 (SAR_RS09620)
**hemE**	(MW2-BA000033-[1928290:1929327])	SAUR1977 (SAR_RS09885)
**prsA2**	(MW2-BA000033-[1934731:1935693])	SAUR1985 (SAR_RS09925)
**vraR**	(MW2-BA000033-[1987314:1987943])	SAUR2061 (SAR_RS10305)
**ftnA**	(MW2-BA000033-[1996034:1996534])	SAUR2071 (SAR_RS10355)
**gatC**	(COL-CP000046-[2023183:2023485])	
**A6U340**	(N315-BA000018-[2000746:2000922])	SAUR2117 (SAR_RS10585)
**groL**	(MW2-BA000033-[2100933:2102549])	SAUR2199 (SAR_RS10995)
**yheS**	(MW2-BA000033-[2118912:2120840])	SAUR2217 (SAR_RS11085)
**yedJ**	(MW2-BA000033-[2171330:2171977])	SAUR2269 (SAR_RS11345)
**rpmE**	(MSHR1132-FR821777-[2115853:2116107])	
**murZ**	(MW2-BA000033-[2201005:2202264])	SAUR2307 (SAR_RS11535)
**pyrG**	(MW2-BA000033-[2204438:2206048])	SAUR2310 (SAR_RS11550)
**coaW**	(MW2-BA000033-[2208310:2209113])	SAUR2313 (SAR_RS11565)
**glmM**	(MW2-BA000033-[2245874:2247229])	SAUR2343 (SAR_RS11715)
**cdaA=dacA**	(MW2-BA000033-[2248190:2248999])	SAUR2345 (SAR_RS11725)
**rplM**	(N315-BA000018-[2290241:2290678])	SAUR2407 (SAR_RS12035)
**rpoA**	(MW2-BA000033-[2306516:2307460])	SAUR2415 (SAR_RS12075)

Gene ID	GenBank coordinates	ID used in PubMLST cgMLST scheme
**infA**	(RF122-AJ938182-[2247044:2247262])	
**rplF**	(N315-BA000018-[2301007:2301543])	SAUR2426 (SAR_RS12130)
**Q5HDQ1**	(N315-BA000018-[2361456:2361707])	
**panC**	(MW2-BA000033-[2680143:2680994])	SAUR2808 (SAR_RS14040)
**lqo**	(MW2-BA000033-[2690198:2691694])	SAUR2818 (SAR_RS14090)
**cudT**	(MW2-BA000033-[2700793:2702415])	SAUR2827 (SAR_RS14135)
**gpxA-L2**	(MW2-BA000033-[2710352:2710849])	SAUR2837 (SAR_RS14185)
**estA**	(N315-BA000018-[2717418:2718179])	SAUR2850 (SAR_RS14250)
**arcR**	(MW2-BA000033-[2723477:2724181])	SAUR2852 (SAR_RS14260)
**arcC-L2**	(MW2-BA000033-[2724280:2725221])	SAUR2853 (SAR_RS14265)
**cspC-L3=cspB**	(MSHR1132-FR821777-[2754573:2754773])	
**rsmG=gidB**	(MW2-BA000033-[2815456:2816175])	SAUR2935 (SAR_RS14675)
**mnmG=gidA**	(MW2-BA000033-[2816175:2818052])	SAUR2936 (SAR_RS14680)
**rnpA**	(MW2-BA000033-[2819637:2819984])	SAUR2938 (SAR_RS14690)
**rpmH**	(MRSA252-BX571856-[2902268:2902405])	

2. Strains used for tree construction

Clonal Complex or Sequence Type	Strain	Accession No.
**CC1**	MSSA476	BX571857
**CC1**	MW2	BA000033
**CC1**	TCH70	ACHH
**CC5**	04-02981	CP001844
**CC5**	06BA18369	ARXY
**CC5**	CIG1150	AHVH
**CC5**	ED98	CP001781
**CC5**	JH1	CP000736
**CC5**	JH9	CP000703
**CC5**	Mu3	AP009324
**CC5**	Mu50	BA000017
**CC5**	N315	BA000018
**CC5**	Strain 10388	HE579059
**CC5**	VRS10	AHBT
**CC6**	PFESA1528	FKTB
**CC6**	Strain394	JVIV
**CC7**	Strain 8-2300	CP015646
**CC7**	TCH959	AASB
**CC8**	CIG2018	AHVV
**CC8**	CIG547	AHVQ
**CC8**	COL	CP000046
**CC8**	FPR3757	CP000255
**CC8**	M1	HF937103
**CC8**	MRSA177	AECP
**CC8**	NCTC8325	CP000253
**CC8**	Newman	AP009351
**CC8**	NRS100	CP007539

**CC8**	TCH1516	CP000730
**CC9**	Strain 21334	AGTW
**CC10**	H19ST10	ACSS
**CC12**	KLT6	APFH
**CC12**	Strain 21266	AFTT
**CC15**	CIGC93	AHVD
**CC15**	KPL1828	AZJB
**CC15**	RUH71	NHZV
**CC15**	VCU006	AGTZ
**CC15-ST582**	08-02119-	CP015645
**CC20**	PFESA2437	FKPW
**CC22**	H-EMRSA-15	CP007659
**CC22**	HO5096-0412	HE681097
**CC22**	IS-105	AHLR
**CC22**	Strain 21310	AFNP
**CC25**	Strain 21193	AFEG
**CC30**	68-397	ACJT
**CC30**	Btn1260	ACUU
**CC30**	CIG1605	AHKE
**CC30**	EMRSA16	ADAT
**CC30**	M876	ACJV
**CC30**	MRSA252	BX571856
**CC30**	WBG10049	ACSV
**CC45 agr I**	A9635	ACKI
**CC45 agr I**	CA347	CP006044
**CC45 agr I**	CIG1524	AHVI
**CC45 agr IV caps 5**	BU N22t6	LFNS
**CC45 agr IV caps 8**	CIG290	AIES
**CC45 agr IV caps 8**	Strain 21252	AHJV
**CC49**	Strain 21262	AHJW
**CC49**	Tager 104	CP012409
**CC49**	WT65	CP084107
**CC50**	Strain 6850	CP006706
**CC59**	PM1	BAFA
**CC59**	SA40	CP003604
**CC59**	SA957	CP003603
**CC72**	CN1	CP003979
**CC72**	TCH130	ACHD
**CC80**	11819-97	CP003194
**CC80**	Strain 103564	AHZI
**CC80**	Strain 21333	AHKA
**CC88**	HST105	AZTH
**CC88**	Strain 21343	AHKV
**CC93**	JKD6159	CP002114
**CC96**	KPL1845	AZJA
**CC97**	IS-55	AHLN
**CC97**	Newbould 305	AKYW
**CC97**	PB32	ANIB

**CC101**	SA083	JXIE
**CC121**	93bS9	CP010952
**CC121**	Strain 21269	AFTU
**CC126**	Sa1FB	PATRIC128024396
**CC130**	O11	CP024649
**CC133**	ED133	CP001996
**ST140**	Kenyaseq6547225	ERR1764920
**ST140**	SO-1977	SRR5682128
**CC152**	55-99-44	CP024998
**CC152**	BB155-	LN854556
**CC188**	CUHK HK188	JFFV
**CC188**	Strain 21340	AGTX
**ST291**	SA3-LAU	JHDV
**CC361**	GR1	AJLX
**CC361**	VH221	AOFX
**CC395**	Strain 21202	AGRO
**CC398**	08BA02176	CP003808
**CC398**	112808A	AHZK
**CC398**	S0385	AM990992
**CC398**	S100	AUPV
**CC425**	LGA251	FR821779
**CC479**	Strain 685	LRNB
**CC599**	H11240036	ERR175867
**CC692**	K12S0375	JYGF
**CC705**	RF122	AJ938182
**CC707**	Strain 21235	AFTQ
**CC772**	CO08	AJKD
**CC772**	KTY21	AOCQ
**CC772**	VH60	ALWG
**CC779**	st2344	FGWF
**CC913**	H1772	OFVV
**CC1153**	3688STDY6124889	FQHT
**CC1153**	Dubai M58	CP065857
**CC1290**	015H	FMMV
**CC1464**	ST1464	ANIT
**CC1943**	H105140339	ERR084755
**CC1956**	WT19	CP084892
**CC2972**	SS015	FQIU
**ST2990**	27-G-H	*this study*
**ST2990**	HS-MSSA	VCMW
**ST3268**	TXA	SAMN04362246
**ST3268**	TXB	SAMN04362247
**ST4168**	16CS0209	*this study*
**CC4803**	NN50	BAEA
**ST7687**	01-RR-86	*this study*
**ST7688**	05-RR-90	*this study*
**ST7689**	08-G-E	*this study*
**ST7690**	09-G-F	*this study*

**ST7691**	13-G-52	*this study*
**ST7692**	17-H-61	*this study*
**ST7693**	29-P-01	*this study*
**ST7694**	40-B-50	*this study*
**ST7695**	16CS0212	*this study*
**ST7745**	03-RR-88	*this study*
**ST7746**	07-G-D	*this study*
**ST7747**	12-G-51	*this study*
**ST7748**	15-G-54	*this study*
**ST7749**	18-H-62	*this study*
**ST7750**	26-G-G	*this study*
**ST7751**	28-G-I	*this study*
**ST7752**	30-P-10	*this study*
**ST7753**	32-T-13	*this study*
**ST7754**	39-B-49	*this study*
***S. argenteus* CC1223**	M051	*CCEN*
***S. argenteus* CC1850**	MSHR1132	FR821777
***S. argenteus* CC2198**	M260	CCEF
***S. argenteus* CC2250**	F87619	JGHK
***S. argenteus* CC2250**	M21126	JGMK
***S. argenteus* CC2596**	H115100079	CCEP
***S. argenteus* CC4587**	JABA32044V6S1	CCEE
***S. roterodami* ST3963**	BDS-54	CP092782
***S. roterodami* ST4326**	BDS-53E	CP092783
***S. roterodami/singaporensis* ST6107**	SS60	JABWHF
***S. roterodami/singaporensis* ST6109**	SS90	JABWHD
***S. roterodami* ST6999**	EMCR19	CAJGUT
***S. roterodami* ST7342**	Zoo28	CP092781.1
***S. schweitzeri* CC2059**	FSCB1B	CCEG
S. schweitzeri **ST (206 303 253 142 196 202 197)**	FSA090	CCEO
***S. schweitzeri* ST1872**	FSA037	CCEH
***S. schweitzeri* ST2022**	FSA084	CCEL
***S. schweitzeri* ST2058**	FSCB5	CCEQ
***S. schweitzeri* ST2074**	FSA096	CCEK
