# Supplementary material for: Sequence Analysis of Novel Staphylococcus aureus Lineages from Wild and Captive Macaques
Source: Int J Mol Sci. 2022 Sep 23;23(19):11225. doi: 10.3390/ijms231911225 (PMC9570271; doi:10.3390/ijms231911225)
Supplement: Supplementary file 1 [file ijms-23-11225-s001.zip › Supplemental File S4_Major genomic islands in study strains.pdf]

**Sequence type, (Isolate ID)**	**GI No. 1 upstream dusC, approx. pos. 38,000**	**GI No. 2 downstream dusC, approx. pos. 38,000**	**GI No. 3, approx. pos. 40-80,000**	**GI No. 4, approx. pos. 170,000,**	**GI No. 5, approx. pos. 280,000**	**GI No. 6 (ssl/lpl), approx. position 400,000**	**GI No. 7, approx. pos. 900,000**	**GI No. 8, pos. 1,500,000**	**GI No. 9 (egc and lukD/E) approx. pos. 1,800,000**	**GI No. 10 (sspP/sspS), approx. pos. 2,000,000**	**GI No. 11, approx. pos. 2,500,000**	**GI No. 12, approx. pos. 2,530,000**	**GI No. 13 (st´xanthin), approx. position 2,650,000**	**GI No. 14, approx. pos. 2,770,000**
**ST2990 (27-G-H)**	Q7A899, dusC-trnc, DUF81-GI, cstR/A/B-GI, sqr, yxiE2	Q6GKK6, Q7A890	lpl, hysA. 5 copies of lpl	Q5HJH7, Q9RL82, A6QDI2	Q6GK23, Q6GK22, Q6GK21, Q2G192, Q6GK19, Q6GK18, Q6GK17, Q9L3N6, DUF600, a 2nd copy of essC, 2 copies of DUF600, Q1Y4R3, DUF600, ADI96784, DUF4064, DUF5084, 2 copies of DUF600	ssl01, ssl02, ssl03, ssl04, ssl05, ssl06, ssl07, ssl08, ssl09, ssl10, hsdM/S-ssl, ssl11, slaP, Q2G0X2	appA, appD, appF, appB, appC	negative	Q5HEX7, A5ITW8, Q6GFQ3, DUF955, Q5HEX3, fstAT, AJP22988, DUF1433, DUF1433, Q6GFP5, A0A0E1X646, Q6GFP3, hsdS/M-spl, splF, splE, splD1, splD2-1, splD2-2, splC, splA, A6QHZ7, ear2, sprB, epiG, A5ITY2, A5ITY3, lukD/E, sprC1, Q2FXA9, Q2FXA8, Q7A4X2	sspP/sspS	teg131, 3 copies of lplsauUSI, nudix	A8YYP8, sasG, sarT, sarU	cbiQ, cbiO, UPF0397, DUF62	cna
**ST3268 (Ma2/A14043)**	mcrB, mcrC, tnp_IS1, tnp_IS232, Q6GD44, dusC-trnc, DUF81-GI, cstR/A/B-GI, sqr	Q6GKK6, ycjY, G7ZTC1, Q2YUT3	3 copies of lpl	Q5HJH7-trnc	Transposon with the penicillinase operon, followed by 3copies of DUF600	ssl01, ssl02, ssl03, ssl04, ssl05, ssl06, ssl07, ssl08, ssl09, ssl10, hsdM/S-ssl, ssl11, slaP, Q2G0X2, lpl7, istB2_IS232, tnp_IS232	appA, appD, appF, appB, appC	Q2YY95, sagD, sagC, nfnB, D9RHF6, Q2YYD5, D9RHF8, stsA, tnp_IS232, tnp_IS200	Q5HEX7, A5ITW8, Q6GFQ3, DUF955, Q5HEX3, DUF1433, hysA, DUF1433, ERS140266_RS01930, hsdS/M-spl, ear2, sprB, epiG, A5ITY2, A5ITY3, lukD/E, sprC1, Q2FXA9, Q2FXA8, Q99T51, A6U2S7, Q7A4X2, seg/n/u/i/m/o	sspP/sspS	teg131, sau96I, dcm_Sau96I	A8YYP8, sasG, sarT, sarU	cbiQ, cbiO, UPF0397, DUF62	negative
**ST4168 (16CS0209)**	tnp_ISSha1, C2G7A0, Q6GKL3, C2G798, UPI0001C11B31, Q6GKL1, tnp_IS232, Q6GD44	Q6GKK6, Q7A890	lpl	Q5HJH7-trnc	A0A0E0VL30, DUF5082, UPI0001C11B8A, Q1Y4R2, Q6GK19, DUF5085, Q1Y4R3, 2 copies of DUF600, Q1Y4R2, Q1Y4R3, Q9L3N6, 4 copies of DUF600	ssl01, ssl02, ssl03, ssl05, ssl07, ssl09, ssl10, hsdM/S-ssl, ssl11, slaP, Q2G0X2, 4 copies of lpl, lipC3,	oppC-GI-RF122, oppD-GI-RF122, oppF-GI-RF122, oppA-GI-RF122	Q931R4	Q5HEX7, A5ITW8, Q5HEX3, sprA, hsdS/M-spl, splF, splE, splD2, splC/B/A, ear2, epiG, ERS140266_RS14190, epiF, epiD, epiC, epiB, epiA, lukD/E, sprC1, Q6GFN4, tnp_IS1, Q7A4X2, seg/n/u/i/m/o	sspP/sspS	teg131	A8YYP8, sasG, sarT, sarU	cbiQ, cbiO, UPF0397, DUF62	cna
**ST7687 (01-RR-86)**	Q7A899, dusC-trnc, DUF81-GI, cstR/A/B-GI, sqr, yxiE2 (as in CC5/8)	Q6GKK6, Q7A890	3 copies of lpl	Q5HJH7, Q9RL82, A6QDI2	A0A0E0VL30, DUF5082, UPI0001C11B8A, Q6GK19, DUF5085, Q1Y4R3, Q9L3N6, DUF600, Q1Y4R2, 3 copies of DUF600	ssl01, ssl02, ssl03, ssl04, ssl05, ssl06, ssl07, ssl08, ssl09, ssl10, hsdM/S-ssl, ssl11, slaP, Q2G0X2, 6 copies of lpl, lipC3	appA, appD, appF, appB, appC	Q2YY95, sagD, sagC, nfnB, D9RHF6, Q2YYD5, D9RHF8, stsA	Q5HEX7, A5ITW8, DUF955, Q5HEX3, DUF1433, sprA, AJP22988, 3 copies of DUF1433, hsdS/M-spl, splF, splC/B/A, A6QHZ7, ear2, sprB, epiG, A5ITY2, A5ITY3, lukD/E, sprC1, Q2FXA9, Q2FXA8, Q7A4X2	sspP/sspS	teg131, Q5HD68, D2NA72, lipC3, 3 copies of lpl, sauS0385, dcm_SauS0385	A8YYP8, sasG, sarT, sarU	cbiQ, cbiO, UPF0397, DUF62	negative
**ST7688 (05-RR-90)**	Q7A899, dusC-trnc, DUF81-GI, cstR/A/B-GI, sqr, yxiE2	Q5HJT2, Q6GD34, A6QD75, A6QD76, A8YZ18, Q6GKK6, ycjY, G7ZTC1, Q7A890, Q2YUT3	3 copies of lpl	Q5HJH7, Q9RL82, A6QDI2	Q6GK23, Q6GK22, Q6GK21, Q2G192, Q6GK19, Q6GK18, Q6GK17, Q9L3N6, Q1Y4R2, Q1Y4R3, and 8 copies of DUF600	ssl01, ssl02, ssl03, ssl04, ssl05, ssl07, ssl08, ssl09, ssl10, hsdM/S-ssl, ssl11, slaP, Q2G0X2, 3 copies of lpl, tnp_IS232, istB2_IS232, 3 copies of lpl, lipC3_SAV0446	appA, appD, appF, appB, appC	D9RHF6, Q2YYD5, D9RHF8, stsA	Q5HEX7, A5ITW8, Q6GFQ3, DUF955, 4 copies of DUF1433, hysA, Q5HE01, hsdS/M-spl, splF, splE, splD1, splC/B/A, A6QHZ7, ear2, sprB, epiG, A5ITY2, A5ITY3, lukD, lukE	sspP/sspS	teg131, 2 copies of lpl, Q5HD68, D2NA72, lipC3, 2 copies of lpl, dcm_Sau3AI, sau3AI	A8YYP8, sasG, sarT, sarU	cbiQ, cbiO, UPF0397, DUF62	negative
**ST7689 (08-G-E)**	Q6GD44	Q6GKK6, Q7A890	2 copies of lpl, lipC3	Q5HJH7, Q9RL82, A6QDI2	esxC, esxB, esaE, esxD, essD, Q1Y4R2, Q1Y4R3, 3 copies of DUF600, Q1Y4R2, Q1Y4R3, 2 copies of DUF600, ADI96784, DUF4064, DUF5084, 3 copies of DUF600	ssl01, ssl02, ssl03, ssl04, ssl05, ssl07, ssl08, ssl09, ssl10, hsdM/S-ssl, ssl11, slaP, Q2G0X2, 6 copies of lpl, lipC3	appA, appD, appF, appB, appC	negative	Q5HEX7, A5ITW8, Q6GFQ3, DUF955, Q5HEX3, tnp_IS3, sprA, DUF1433, Q6GFP5, A0A0E1X646, hsdS/M-spl, splF, splD1, splC/B/A, A6QHZ7, ear2, sprB, A5ITY2, lukD/E, Q2FXA9, Q2FXA8, Q99T51, A6U2S7, Q7A4X2, seg/n/u/i/m/o	sspP/sspS	teg131, 2 copies of lpl, Q5HD68, D2NA72, lipC3, 3 copies of lpl, sauUSI, nudix	A8YYP8, sasG, sarT, sarU	cbiQ, cbiO, UPF0397, DUF62	negative
**ST7690 (09-G-F)**	Q7A899, dusC-trnc, DUF81-GI, cstR/A/B-GI, sqr, yxiE2	Q5HJT2, Q6GD34, A6QD75, A6QD76, A8YZ18, Q6GKK6, ycjY, tnp-Q6GH59, tnp-Q6GJF3, Q7A890	3 copies of lpl, hysA, 3 copies of lpl	Q5HJH7, tnp-Q6GH59, tnp-Q6GJF3	Q6GK23, Q6GK22, Q6GK21, Q2G192, Q6GK19, Q6GK18, Q6GK17, Q9L3N6, 4 copies of DUF600, Q1Y4R2, Q1Y4R3, 4 copies of DUF600	ssl01, ssl02, ssl03, ssl04, ssl05, ssl07, ssl08, ssl09, ssl10, hsdM/S-ssl, ssl11, slaP, Q2G0X2, 6 copies of lpl, lipC3	appA, appD, appF, appB, appC	negative	Q6GFQ3, DUF955, Q5HEX3, istB2_IS232, tnp_IS232, sprA, DUF1433, Q6GFP5, A0A0E1X646, hsdS/M-spl, splF, splE, splC/B/A, A6QHZ7, ear2, sprB, epiG, A5ITY2, A5ITY3, lukD/E, sprC1, Q2FXA9, Q2FXA8, Q7A4X2	sspP/sspS	teg131, 4 copies of lpl istB2_IS232, tnp_IS232, Q5HD68, D2NA72, lipC3, lpl, sauS0385, dcm_SauS0385	A8YYP8, sasG, sarT, sarU	cbiQ, cbiO, UPF0397, DUF62	cna
**ST7691 (13-G-52)**	Q6GD44	Q6GKK6, Q7A890	2 copies of lpl, lipC3	Q5HJH7, Q9RL82, A6QDI2	esxC, esxB, esaE, esxD, essD, TIGR01741, Q1Y4R2, Q1Y4R3, DUF600	ssl01, ssl02, ssl03, ssl04, ssl05, ssl06, ssl07, ssl08, ssl09, ssl10, hsdM/S-ssl, ssl11, slaP, Q2G0X2, 6 copies of lpl, lipC3	appA, appD, appF, appB, appC	negative	Q6GFQ3, DUF955, Q5HEX3, tnp_IS3, sprA, DUF1433, Q6GFP5, A0A0E1X646, hsdS/M-spl, splF, splD1, splC/B/A, A6QHZ7, ear2, sprB, A5ITY2, lukD/E, Q2FXA9, Q2FXA8, Q99T51, A6U2S7, Q7A4X2, seg/n/u/i/m/o	sspP/sspS	teg131, lpl, Q5HD68, D2NA72, lipC3, 3 copies of lplsauUSI, nudix	A8YYP8, sasG, sarT, sarU	cbiQ, cbiO, UPF0397, DUF62	cna
**ST7692 (17-H-61)**	Q6GD44	Q5HJT2, Q6GD34, A6QD75, A6QD76, A8YZ18, Q6GKK6, ycjY, G7ZTC1, Q7A890, Q2YUT3	4 copies of lpl	Q5HJH7, Q9RL82, A6QDI2	Q6GK23, Q6GK22, Q6GK21, Q2G192, Q6GK19, Q6GK18, Q9L3N6, 2 copies of DUF600, Q1Y4R3, DUF600	ssl01, ssl02, ssl03, ssl04, ssl05, ssl07, ssl08, ssl09, ssl10, hsdM/S-ssl, ssl11, slaP, Q2G0X2, 5 copies of lpl, lipC3	appA, appD, appF, appB, appC	Q2YY95, sagD, sagC, nfnB, D9RHF6, Q2YYD5, D9RHF8, stsA	Q5HEX7, A5ITW8, Q6GFQ3, DUF955, Q5HEX3, AJP22988, DUF1433, DUF1433, DUF1433, DUF1433, hysA, A0A0E1X646, hsdS/M-spl, splF, splE, splD1, splC/B/A, A6QHZ7, ear2, sprB, epiG, A5ITY2, A5ITY3, lukD/E, A6U2S7, Q7A4X2, Q99T51, int-2b	sspP/sspS	teg131, 4 copies of lpl Q5HD68, D2NA72, lipC3_SACOL2496, 2 copies of lpl dcm_Sau3AI, sau3AI	A8YYP8, sasG, sarT, sarU	cbiQ, cbiO, UPF0397, DUF62	negative
**ST7693 (29-P-01)**	negative	Q6GKK6, ycjY, G7ZTC1, Q7A890, Q2YUT3	lpl	Q5HJH7, Q9RL82, A6QDI2	esxC, esxB, esaE, esxD, essD, TIGR01741, Q1Y4R2, Q1Y4R3, DUF600	ssl01, ssl02, ssl03, ssl04, ssl05, ssl07, ssl08, ssl09, ssl10, hsdM/S-ssl, ssl11, slaP, Q2G0X2, 6 copies of lpl, lipC3	appA, appD, appB, appC	negative	Q6GFQ3, DUF955, Q5HEX3, sprA, DUF1433, Q6GFP5, A0A0E1X646, hsdS/M-spl, splF, splE, splD1, splC/B/A, A6QHZ7, ear2, sprB, A5ITY2, lukD/E, A6U2S7, Q7A4X2, Q99T51, int-2b	sspP/sspS sspP/sspS	teg131, Q5HD68, D2NA72, lipC3, lpl, tx_universal3, dcm_SauRF122, sauRF122, zoo28neu_002509	Q5HJU6, sarT, sarU	cbiQ, cbiO, UPF0397, DUF62	negative
**ST7694 (40-B-50)**	negative	Q6GKK6, ycjY, G7ZTC1, Q7A890, Q2YUT3	5 copies of lpl	Q5HJH7-trnc	esxC, esxB, esaE, esxD, essD, TIGR01741, Q1Y4R2, Q1Y4R3, DUF600	ssl01, ssl02, ssl03, ssl04, ssl05, ssl07, ssl08, ssl09, ssl10, hsdM/S-ssl, ssl11, slaP, Q2G0X2	appA, appD, appF, appB, appC	D9RHF6, Q2YYD5, D9RHF8, stsA	Q5HEX7, A5ITW8, Q6GFQ3, DUF955, Q5HEX3, sprA, DUF1433, Q6GFP5, A0A0E1X646, hsdS/M-spl, splF, splD1, splC/B/A, A6QHZ7, ear2, sprB, epiG, A5ITY2, A5ITY3, lukD/E, sprC1, Q2FXA9, Q2FXA8, Q7A4X2	sspP/sspS	teg131, 4 copies of lpl sauUSI, nudix	A8YYP8, sasG, sarT, sarU	cbiQ, cbiO, UPF0397, DUF62	negative
**ST7695 (16CS0212)**	Q6GKL6	Q5HJT2, Q6GD34, A6QD75, A6QD76, A8YZ18, Q6GKK6, ycjY, G7ZTC1	lpl	Q5HJH7, Q9RL82, A6QDI2	esxC, esxB, esaE, esxD, essD, Q1Y4R2, Q1Y4R3, DUF600, Q1Y4R2, Q1Y4R3, Q9L3N6, DUF600, ADI96784, DUF4064, DUF5084	ssl01, ssl02, ssl04, ssl05, ssl07, ssl08, ssl09, ssl10, hsdM/S-ssl, ssl11, slaP, Q2G0X2, 5 copies of lpl, lipC3	appA, appD, appF, appB, appC	negative	Q5HEX7, A5ITW8, sprA, DUF1433, hysA, DUF1433, A0A0E1X646, hsdS/M-spl, splF, splD2, splE, splC/B/A, ear2, epiG, ERS140266_RS14190, epiE/F/P/D/C/B/A, bsaX, lukD/E, sprC1, Q2FXA9, Q2FXA8	sspP/sspS	teg131, BDS-53E_002366, lpl, Q5HD68, D2NA72, lipC3, 2 copies of lpl dcm_Sau3AI, sau3AI	negative	negative	negative

Sequence type, (Isolate ID)	GI No. 1 upstream *dusC*, approx. pos. 38,000	GI No. 2 downstream *dusC*, approx. pos. 38,000	GI No. 3, approx. pos. 40-80,000	GI No. 4, approx. pos. 170,000,	GI No. 5, approx. pos. 280,000	GI No. 6 (*ssl*/*lpl*), approx. position 400,000	GI No. 7, approx. pos. 900,000	GI No. 8, pos. 1,500,000	GI No. 9 (*egc* and *lukD/E*) approx. pos. 1,800,000	GI No. 10 (*sspP/sspS*), approx. pos. 2,000,000	GI No. 11, approx. pos. 2,500,000	GI No. 12, approx. pos. 2,530,000	GI No. 13 (st´xanthin), approx. position 2,650,000	GI No. 14, approx. pos. 2,770,000
**ST7745 (03-RR-88)**	*lrpC*, Q6GD44	Q6GKK6, Q7A890	5 copies of *lpl*	Q5HJH7, Q9RL82, A6QDI2	Q6GK23, Q6GK22, Q6GK21, Q6GK19, Q6GK18, Q6GK17, Q9L3N6, TIGR01741, 2 copies of DUF600, ADI96784, DUF4467, DUF4064, DUF5084, DUF600	ssl01, ssl02, ssl03, ssl04, ssl05, ssl06, ssl07, ssl08, ssl09, ssl10, hsdM/S-ssl, ssl11, slaP, Q2G0X2, *lpl_locus-0450,* tnp_IS232, istB2_IS232, *lipC3*_SAA6159_00398	*appA, appD, appF, appB, appC*	Q2YY95, *sagD, sagC, nfnB,* D9RHF6, Q2YYD5, D9RHF8, *stsA*	Q5HEX7, A5ITW8, Q6GFQ3, DUF955, DUF1433, *sprA*, AJP22988, 2 copies of DUF1433, hsdS/M-spl, splF, splE, splC/B/A, A6QHZ7, ear2, *sprB*, *epiG*, A5ITY2, A5ITY3, *lukD/E*, sprC1, Q2FXA9, Q2FXA8, Q7A4X2	*sspP/sspS*	teg131, Q5HD68, D2NA72, *lipC3*, *lpl*, dcm_SauRF122, sauRF122	negative	*cbiQ, cbiO*, UPF0397, DUF62	*cna*
**ST7746 (07-G-D)**	Q7A899, *dusC*-trnc, DUF81-GI, *cstR/A/B*-GI, *sqr, yxiE2*	Q5HJT2, Q6GD34, A6QD75, A6QD76, A8YZ18, Q6GKK6, *ycjY*, G7ZTC1, Q7A890	6 copies of *lpl*	A5IP65, A5IP66, A5IP67, A5IP68, A5IP69, A5IP70, A5IP71, A5IP72, A6TXY8, A5IP73, A6QDI2	A0A0Z1N098, A0A0U0VFZ6, A0A122YW60, ADI96784, DUF4064, DUF5084, Q1Y4R2, Q1Y4R3, Q9L3N6, 2 copies of DUF600, Q1Y4R2, Q1Y4R3, 2 copies of DUF600	ssl01, ssl02, ssl03, ssl04, ssl05, ssl06, ssl07, ssl08, ssl09, ssl10, hsdM/S-ssl, ssl11, slaP, Q2G0X2	*appA, appD, appF, appB, appC*	negative	DUF1433, *hysA*, 2 copies of DUF1433, Q6GFP5, A0A0E1X646, hsdS/M-spl, splF, splE, splD1, splC/B/A, A6QHZ7, ear2, *sprB*, *epiG*, A5ITY2, A5ITY3, *lukD/E*, sprC1, Q2FXA9, Q2FXA8, Q7A4X2	*sspP/sspS*	teg131, 2 copies of *lpl*, Q5HD68, D2NA72, *lipC3*, *lpl*, tnp_IS232, istB2_IS232, 2 copies of *lpl*, D2NA72, *lipC3*, *lpl*, sauS0385, dcm_SauS0385	negative	*cbiQ, cbiO*, UPF0397, DUF62	*cna*
**ST7747 (12-G-51)**	H0CD41, D2N3D1, C2G7A0, Q6GKL3, C2G798, UPI0001C11B31, D2N3E0, Q6GKL1, tnp_IS232, Q6GD44<br>*Similar as in CC398 (except for some tnps)*	Q6GKK6, Q7A890	*lpl*, *hysA*, 2 copies of *lpl*, istB2_IS232, tnp_IS232, *lpl*	Q5HJH7, Q9RL82, A6QDI2	A0A0E0VL30, DUF5082, UPI0001C11B8A, Q1Y4R2, Q6GK19, DUF5085, Q1Y4R3, Q9L3N6, DUF600, Q1Y4R2, Q1Y4R3, 3 copies of DUF600	ssl01, ssl02, ssl03, ssl04, ssl05, ssl07, ssl08, ssl09, ssl10, hsdM/S-ssl, ssl11, slaP, Q2G0X2, istB2_IS232, tnp_IS232, 3 copies of *lpl*	*appA, appD, appF, appB, appC*	Q2YY95, *sagD, sagC, nfnB,* D9RHF6, Q2YYD5, D9RHF8, *stsA*	Q5HEX7, A5ITW8, Q6GFQ3, DUF955, Q5HEX3, DUF1433, *hysA*, hsdS/M-spl, splF, splE, splD1, splC/B/A, A6QHZ7, ear2, *sprB*, *epiG*, ERS140266_RS14190, epiE/F/P/D/C/B/A, bsaX, *epiA*, *lukD/E*, Q2YTQ0, Q6GFN4, tnp_IS1, A6U2S7, Q7A4X2, Q99T51	*sspP/sspS*	teg131, *lpl*, Q5HD68, D2NA72, *lipC3*, *lpl*, sauS0385, dcm_SauS0385	A8YYP8	negative	negative
**ST7748 (15-G-54)**	Q6GD44	Q6GKK6, Q7A890	2 copies of *lpl*, *lipC3*	Q5HJH7, Q9RL82, A6QDI2	*esxC, esxB, esaE, esxD, essD,* TIGR01741, Q1Y4R2, Q1Y4R3, DUF600	ssl01, ssl02, ssl03, ssl04, ssl05, ssl07, ssl08, ssl09, ssl10, hsdM/S-ssl, ssl11, slaP, Q2G0X2, 6 copies of *lpl*, *lipC3*	*oppB-GI-RF122, oppC-GI-RF122, oppD-GI-CIG93, oppF-GI-RF122, oppA-GI-RF122*	negative	Q5HEX7, A5ITW8, Q6GFQ3, DUF955, Q5HEX3, tnp_IS3, tnp_IS3, r122, *sprA*, DUF1433, Q2FXD2, hsdS/M-spl, splF, splE, splD1, splC/B/A, A6QHZ7, ear2, *sprB*, A5ITY2, *lukD/E*, Q2FXA9, Q2FXA8, Q99T51, A6U2S7, Q7A4X2, *seg/n/u/i/m/o*	*sspP/sspS*	teg131, BDS-53E_002366, *lpl*, Q5HD68, D2NA72, *lipC3*, 2 copies of *lpl,* dcm_Sau3AI, sau3AI	Q5HJU6, sarT, sarU	*cbiQ, cbiO*, UPF0397, DUF62	*cna*
**ST7749 (18-H-62)**	hsdM5, hsdR5, Q6GKL3, C2G798, D2N3E0, Q6GKL1, tnp_IS232, Q6GD44	Q6GKK6, Q7A890	3 copies of *lpl*, *hysA*, 3 copies of *lpl*	Q5HJH7, Q9RL82, A6QDI2	Q6GK23, Q6GK22, Q6GK21, Q2G192, Q6GK19, Q6GK18, Q6GK17, Q9L3N6, 2 copies of DUF600, Q1Y4R2, Q1Y4R3, 2 copies of DUF600	ssl01, ssl02, ssl03, ssl04, ssl05, ssl07, ssl08, ssl09, ssl10, hsdM/S-ssl, ssl11, slaP, Q2G0X2, 6 copies of *lpl*, *lipC3*	*appA, appD, appF, appB, appC*	D9RHF6, Q2YYD5, D9RHF8, *stsA*	Q5HEX7, A5ITW8, Q6GFQ3, DUF955, Q5HEX3, *sprA*, DUF1433, Q6GFP5, A0A0E1X646, hsdS/M-spl, splF, splC/B/A, A6QHZ7, ear2, *sprB*, *epiG*, ERS140266_RS14190, epiE/F/P/D/C/B/A, *lukD/E*, sprC1, Q2FXA9, Q2FXA8, Q7A4X2	*sspP/sspS*	teg131, BDS-53E_002366, 2 copies of *lpl* Q5HD68, D2NA72, *lipC3*_SACOL2496, 2 copies of *lpl* dcm_Sau3AI, sau3AI	A8YYP8	*cbiQ, cbiO*, UPF0397, DUF62	negative
**ST7750 (26-G-G)**	H0CD41, D2N3D1, Q6GD44	Q6GKK6	3 copies of *lpl*, *lipC3*	A5IP65, A5IP66, A5IP67, A5IP68, A5IP69, A5IP70, A5IP71, A5IP72, A6TXY8, A5IP73	Q6GK23, Q6GK22, Q6GK21, Q2G192, Q6GK19, Q6GK18, Q6GK17, Q9L3N6, 3 copies of DUF600, ADI96784, DUF4467, DUF4064, DUF5084, 4 copies of DUF600	ssl01, ssl02, ssl03, ssl04, ssl05, ssl07, ssl08, ssl09, ssl10, hsdM/S-ssl, ssl11, slaP, Q2G0X2, 3 copies of *lpl*, *lipC3*	*appA, appD, appF, appB, appC*	Q2YY95, *sagD, sagC, nfnB,* D9RHF6, Q2YYD5, D9RHF8, *stsA*	DUF1433, *hysA*, DUF1433, DUF1433, Q6GFP5, A0A0E1X646, hsdS/M-spl, splF, splE, splC/B/A, A6QHZ7, ear2, *sprB*, *epiG*, A5ITY2, A5ITY3, *lukD/E*, sprC1, Q2FXA9, Q2FXA8, Q7A4X2	*sspP/sspS*	teg131, BDS-53E_002366, 2 copies of *lpl* Q5HD68, D2NA72, *lipC3*, 2 copies of *lpl,* dcm_Sau3AI, sau3AI	A8YYP8, *sasG, sarT, sarU*	*cbiQ, cbiO*, UPF0397, DUF62	*cna*
**ST7751 (28-G-I)**	C2G7A0, Q6GKL3, C2G798, UPI0001C11B31, D2N3E0, Q6GKL1, Q6GD44	Q6GKK6, *ycjY*, G7ZTC1, Q7A890	*lpl*, *hysA*. 4 copies of *lpl*	Q5HJH7, Q9RL82, A6QDI2	*esxC, esxB, esaE, esxD, essD,* Q1Y4R2, Q1Y4R3, Q9L3N6, 2 copies of DUF600, Q1Y4R2, Q1Y4R3, TIGR01741, DUF600	ssl01, ssl02, ssl03, ssl04, ssl05, ssl07, ssl08, ssl09, ssl10, hsdM/S-ssl, ssl11, slaP, Q2G0X2, 3copies of *lpl*	*appA, appD, appF, appB, appC*	negative	Q5HEX7, A5ITW8, DUF955, Q5HEX3, DUF1433, *sprA*, DUF1433, DUF1433, hsdS/M-spl, splF, splE, splD1, splC/B/A, A6QHZ7, ear2, *sprB*, *epiG*, A5ITY2, A5ITY3, *lukD/E*, Q2FXA9, Q2FXA8, Q7A4X2	*sspP/sspS*	teg131, *lpl*, Q5HD68, D2NA72, *lipC3*, 3 copies of *lpl*dcm_Sau3AI, sau3AI	Q5HJU6, sarT, sarU	*cbiQ, cbiO*, UPF0397, DUF62	negative
**ST7752 (30-P-10)**	Q7A899, *dusC*-trnc, DUF81-GI, *cstR/A/B*-GI, *sqr, yxiE2*	Q5HJT2, Q6GD34, A6QD75, A6QD76, A8YZ18, Q6GKK6, *ycjY*, G7ZTC1	5 copies of *lpl*	Q5HJH7, Q9RL82, A6QDI2	A0A0Z1N098, A0A0U0VFZ6, A0A122YW60, ADI96784, DUF4064, DUF5084, Q1Y4R2, Q1Y4R3, Q9L3N6, 2 copies of DUF600, Q1Y4R2, Q1Y4R3, 4 copies of DUF600	ssl01, ssl02, ssl03, ssl04, ssl05, ssl06, ssl07, ssl08, ssl09, ssl10, hsdM/S-ssl, ssl11, slaP, Q2G0X2, 5 copies of *lpl, lipC3*	*appA, appD, appF, appB, appC*	negative	Q5HEX7, A5ITW8, Q6GFQ3, DUF955, Q5HEX3, fstAT, AJP22988, DUF1433, DUF1433, Q6GFP5, A0A0E1X646, hsdS/M-spl, splF, splE, splD1, splC/B/A, A6QHZ7, ear2, *sprB*, *epiG*, A5ITY2, A5ITY3, *lukD/E*	*sspP/sspS*	teg131, *lpl*-locus-2500, dcm_Sau3AI, sau3AI	A8YYP8	*cbiQ, cbiO*, UPF0397, DUF62	negative
**ST7753 (32-T-13)**	*mcrC, dhlC*-trnc, Q6GD44	Q6GKK6, Q7A890	6 copies of *lpl*	Q5HJH7, Q9RL82, A6QDI2	*esxC, esxB, esaE, esxD, essD,* Q1Y4R2, Q1Y4R3, 3 copies of DUF600, Q1Y4R2, Q1Y4R3, 2 copies of DUF600, ADI96784, DUF4064, DUF5084, 3 copies of DUF600	ssl01, ssl02, ssl03, ssl04, ssl05, ssl07, ssl08, ssl09, ssl10, hsdM/S-ssl, ssl11, slaP, Q2G0X2, tnp_IS232, istB2_IS232, 4 copies of *lpl*,	*appA, appD, appF, appB, appC*	D9RHF6, Q2YYD5, D9RHF8, *stsA*	Q6GFQ3, DUF955, Q5HEX3, *sprA*, DUF1433, Q6GFP5, A0A0E1X646, hsdS/M-spl, splF, splD1, splC/B/A, A6QHZ7, ear2, *sprB*, A5ITY2, *lukD/E*, Q2FXA9, Q2FXA8, Q99T51, A6U2S7, Q7A4X2, *seg/n/u/i/m/o*	*sspP/sspS*	teg131, Q5HD68, D2NA72, *lipC3*, *lpl*, tx_universal3, dcm_SauRF122, sauRF122	A8YYP8	*cbiQ, cbiO*, UPF0397, DUF62	*cna*
**ST7754 (39-B-49)**	C1PH94, C1PH95, istB2_IS232, tnp_IS232, lrpC, Q6GD44, tnp_IS1272, DUF81-GI, *cstR/A/B*-GI, *sqr, yxiE2*	Q6GKK6, Q7A890	*lpl*, *lipC3*	Q5HJH7, Q9RL82, A6QDI2	Q6GK23, Q6GK22, Q6GK21, Q2G192, Q6GK19, Q6GK18, Q6GK17, Q9L3N6, 2 copies of DUF600	ssl01, ssl02, ssl03, ssl04, ssl05, ssl07, ssl08, ssl09, ssl10, hsdM/S-ssl, ssl11, slaP, Q2G0X2, 3 copies of *lpl*, *lipC3*	*appA, appD, appF, appB, appC*	Q931R4	Q6GFQ3, DUF955, Q5HEX3, tnp_IS3, tnp_IS3, r122, *sprA*, DUF1433, Q2FXD2, hsdS/M-spl, splF, splE, splD1, splC/B/A, A6QHZ7, ear2, *sprB*, *epiG*, A5ITY2, A5ITY3, *lukD/E*, sprC1, Q2FXA9, Q2FXA8, Q7A4X2	*sspP/sspS*	Q5HD68, D2NA72, *lipC3*, 2 copies of *lpl* sauUSI, nudix	negative	*cbiQ, cbiO*, UPF0397, DUF62	*cna*
